# Distinct Signaling by Ventral Tegmental Area Glutamate, GABA, and Combinatorial Glutamate-GABA Neurons in Motivated Behavior

**DOI:** 10.1016/j.celrep.2020.108094

**Published:** 2020-09-01

**Authors:** David H. Root, David J. Barker, David J. Estrin, Jorge A. Miranda-Barrientos, Bing Liu, Shiliang Zhang, Hui-Ling Wang, Francois Vautier, Charu Ramakrishnan, Yoon Seok Kim, Lief Fenno, Karl Deisseroth, Marisela Morales

**Affiliations:** 1 Neuronal Networks Section, Integrative Neuroscience Research Branch, National Institute on Drug Abuse, 251 Bayview Boulevard Suite 200, Baltimore, MD 21224, USA; 2 Confocal and Electron Microscopy Core, National Institute on Drug Abuse, 251 Bayview Boulevard Suite 200, Baltimore, MD 21224, USA; 3 National Institute on Drug Abuse Intramural Research Program, 251 Bayview Boulevard Suite 200, Baltimore, MD 21224, USA; 4 Department of Bioengineering, Stanford University, Stanford, CA 94305, USA; 5 Department of Psychiatry and Behavioral Sciences, Stanford University, Stanford, CA 94305, USA; 6 Howard Hughes Medical Institute, Stanford University, Stanford, CA 94305, USA; 7 Present address: Department of Psychology and Neuroscience, University of Colorado, 2860 Wilderness Place, Boulder, CO 80301, USA; 8 Present address: Department of Psychology, Rutgers University, 152 Frelinghuysen Road, Piscataway, NJ 08854, USA; 9 These authors contributed equally; 10 Lead Contact

## Abstract

Ventral tegmental area (VTA) neurons play roles in reward and aversion. We recently discovered that the VTA has neurons that co-transmit glutamate and GABA (glutamate-GABA co-transmitting neurons), transmit glutamate without GABA (glutamate-transmitting neurons), or transmit GABA without glutamate (GABA-transmitting neurons). However, the functions of these VTA cell types in motivated behavior are unclear. To identify the functions of these VTA cell types, we combine recombinase mouse lines with INTRSECT2.0 vectors to selectively target these neurons. We find that VTA cell types have unique signaling patterns for reward, aversion, and learned cues. Whereas VTA glutamate-transmitting neurons signal cues predicting reward, VTA GABA-transmitting neurons signal cues predicting the absence of reward, and glutamate-GABA co-transmitting neurons signal rewarding and aversive outcomes without signaling learned cues related to those outcomes. Thus, we demonstrate that genetically defined subclasses of VTA glutamate and GABA neurons signal different aspects of motivated behavior.

## In Brief

Root et al. examine ventral tegmental area (VTA) neurons that release glutamate without GABA, GABA without glutamate, or both glutamate and GABA. Cell types have differential projection densities and unique neuronal activity profiles related to cues predicting rewarding, nonreward, or aversive outcomes and in the receipt of predicted rewards.

## Graphical Abstract

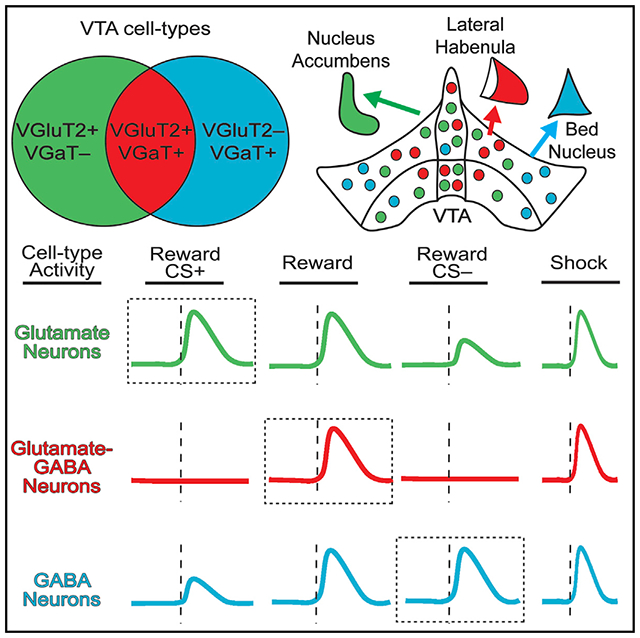

## INTRODUCTION

The ventral tegmental (VTA) area has been traditionally considered a dopaminergic structure that plays important roles in motivated behavior, but accumulating evidence indicates that VTA dopamine neurons are intermingled with neurons that utilize GABA or glutamate as signaling molecules (Barker et al., 2016; Morales and Margolis, 2017; Root et al., 2016). In addition, the VTA has combinatorial neurons that co-release dopamine with either glutamate (Zhang et al., 2015) or GABA (Tritsch et al., 2012) and combinatorial glutamate neurons that co-release glutamate and GABA (Root et al., 2014b, 2018b). The presence of diverse classes of VTA glutamate and GABA neurons within the VTA has been established by neuroanatomical studies, in which glutamatergic neurons have been identified by their expression of vesicular glutamate transporter 2 (VGluT2) (Yamaguchi et al., 2007), GABA neurons by co-expression of glutamic acid decarboxylase (GAD) (an enzyme for GABA synthesis) and vesicular GABA transporter (VGaT), and combinatorial glutamate-GABA neurons by their co-expression of VGluT2, GAD, and VGAT (Root et al., 2018b). Optogenetic behavioral studies have shown that VTA VGaT (Tan et al., 2012) and VGluT2 neurons (Root et al., 2014a; Wang et al., 2015; Yoo et al., 2016) participate in motivated behavior, but it is unclear the extent to which different aspects of motivated behaviors are mediated by neurons that co-transmit glutamate and GABA (VGluT2^+^ VGaT^+^ neurons), release glutamate without GABA (VGluT2^+^ VGaT^−^ neurons), or release GABA without glutamate (VGluT2^−^ VGaT^+^ neurons).

Here, we applied intersectional and subtractive genetic approaches to selectively access VTA VGluT2^+^ VGaT^+^, VGluT2^+^ VGaT^−^, and VGluT2^−^ VGaT^+^ neurons and determined their signaling during reward- and aversion-based behavior. By crossing *Vglut2-Cre* mice with *Vgat-FlpO* mice, we generated dual *Cre/FlpO* transgenic mice that, together with intra-VTA injections of newly developed INTRSECT2.0 (intronic recombinase sites enabling combinatorial targeting; Fenno et al., 2014, 2020), viral vectors allowed the selective targeting of VTA VGluT2^+^ VGaT^+^, VGluT2^+^ VGaT^−^, and VGluT2^−^ VGaT^+^ neurons. By anatomical and electrophysiological analyses, we validated the selective recombination in each class of neurons within the VTA of viral-injected Cre/FlpO mice. Next, by intra-VTA injections in Cre/FlpO mice of INTRSECT2.0 GCaMP6m viral vectors (and after verification of the specific expression of GCaMP6m within the defined classes of neurons), we detected calcium transients (as an indicator of neuronal activity) in response to reward (sucrose), punishment (footshock), or the presentation of learned cues that predicted the delivery of these stimuli.

We found that VTA glutamate-GABA co-transmitting, glutamate-transmitting, and GABA-transmitting neurons increased their activity in response to the presence of reward. However, the glutamate-GABA co-transmitting neurons (VGluT2^+^ VGaT^+^) did not respond to cues predicting reward or cues predicting the absence of reward. In contrast, we observed that glutamate-transmitting neurons (VGluT2^+^ VGaT^−^) increased their neuronal activity in response to cues predicting reward, although GABA-transmitting neurons (VGluT2^−^ VGaT^+^) increased their activity in response to cues predicting the absence of reward. Notably, we also determined that glutamate-GABA co-transmitting neurons were the only cell type to differentially respond to errors in the prediction or omission of reward. We also determined that VTA glutamate-GABA co-transmitting, glutamate-transmitting, and GABA-transmitting neurons all increased their activity in response to an aversive footshock. Nonetheless, although glutamate-GABA co-transmitting neurons did not increase their activity in response to cues predicting footshock, both glutamate-transmitting and GABA-transmitting neurons releasing neurons did increase their activity in response to cues predicting footshock. These findings indicate that, within the total population of VTA VGluT2 and VGaT neurons, there are genetically defined subclasses of neurons that have both overlapping as well as unique responses to specific aspects of motivated behavior. A defining feature of the recently identified VTA glutamate-GABA neurons is their signaling of rewarding or aversive outcomes together with a lack of responsiveness to learned cues predicting these outcomes.

## RESULTS

### Selective Targeting of VTA VGluT2^+^ VGaT^+^, VGluT2^+^ VGaT^−^, and VGluT2^−^ VGaT^+^ Neurons

**Figure 1.**
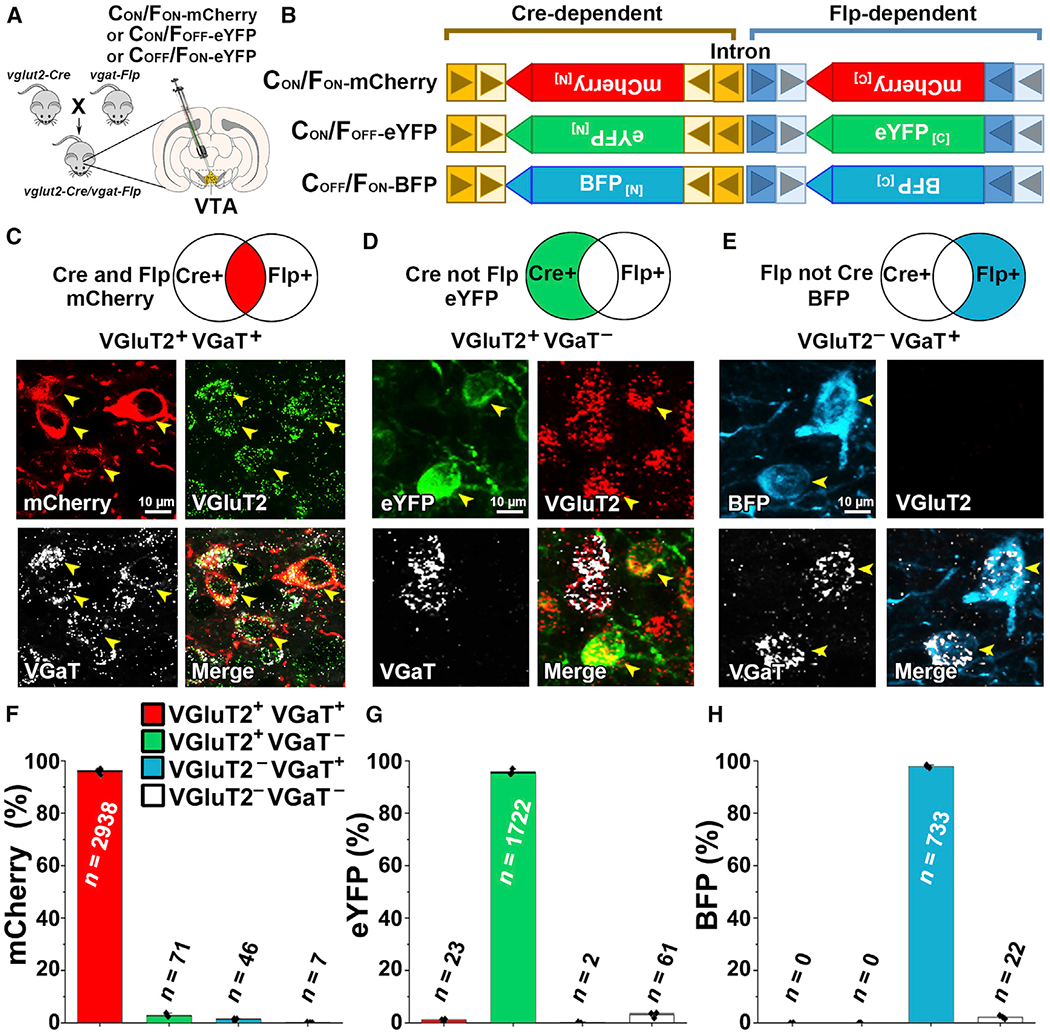
Selective Targeting of VTA VGluT2^+^ VGaT^+^, VGluT2^+^ VGaT^−^, and VGluT2^−^ VGaT^+^ Neurons (A and B) Schematic of crossing of *vglut2-Cre* mice with *vgat-Flp* mice to generate *vglut2-Cre/vgat-Flp* mice and intra-VTA injections of INTRSECT2.0 AAV-C_ON_/F_ON_-mCherry (targeting VGluT2^+^ VGaT^+^ neurons), AAV-C_ON_/F_OFF_-eYFP (targeting VGluT2^+^ VGaT^−^ neurons), or AAV-C_OFF_/F_ON_-BFP vectors (targeting VGluT2^−^ VGaT^+^ neurons). INTRSECT vectors split the fluorophore sequence in half (N or C terminus) between two independent lox sites (boxed Cre-dependent triangles are loxN and lox2722) and FRT sites (boxed Flp-dependent triangles are F3 and F5). Lox and FRT sequences between N and C termini are within introns, which are spliced out for recombination of the entire fluorophore sequence. (C) Co-expression of VGluT2 mRNA and VGaT mRNA in VTA VGluT2^+^ VGaT^+^ mCherry neurons. (D) VGluT2 mRNA without VGaT in VTA VGluT2^+^ VGaT^−^ eYFP neurons. (E) VGaT mRNA without VGluT2 in VTA VGluT2^−^ VGaT^+^ BFP neurons. (F–H) Detection of VGluT2 or VGaT mRNAs within the subpopulations of neurons co-expressing mCherry (F), eYFP (G), or BFP (H). Black dots are individual mice (N = 3/group).

For the targeting of select subpopulations of VTA glutamate and GABA neurons, we generated *vglut2-Cre/vgat-Flp* mice (by crossing *vglut2-Cre* mice with *vgat-Flp* mice; Figure 1A) and injected into the VTA of these mice newly developed INTRSECT2.0 adeno-associated viral (AAV) vectors (Fenno et al., 2020). We used AAV-C_ON_/F_ON_-mCherry vectors requiring both Cre recombinase (henceforth referred to as C_ON_) and Flp recombinase (henceforth referred to as F_ON_) for mCherry expression to target VGluT2^+^ VGaT^+^ neurons; AAV-C_ON_/F_OFF_-eYFP vectors requiring the presence of Cre recombinase and the absence of Flp recombinase (henceforth referred to as F_OFF_) for expression of enhanced yellow fluorescent protein (eYFP) to target VGluT2^+^ VGaT^−^ neurons, and AAV-C_OFF_/F_ON_-blue fluorescent protein (BFP) vector requiring the absence of Cre recombinase (henceforth referred to as C_OFF_) and presence of Flp recombinase for the expression of BFP to target VGluT2^−^ VGaT^+^ neurons (Figure 1B). After confirming VTA neuronal expression of mCherry (Figure 1C), eYFP (Figure 1D), or BFP (Figure 1E), we examined the expression of VGluT2 or VGaT mRNAs within each type of fluorescent VTA neuron (Figures 1C–1E). Within the total population of targeted VGluT2^+^ VGaT^−^-expressing mCherry neurons (3,062 neurons, 3 mice; Figures 1C and 1F), we found that more than 95% expressed both VGluT2 and VGaT mRNAs (95.68% ± 0.64%; 2,938/3,062), about 3% expressed only VGluT2 mRNA (2.85% ± 0.45%; 71/3,062 neurons), close to 1% expressed only VGaT mRNA (1.29% ± 0.23%; 46/3,062), and few lacked VGluT2 and VGaT mRNAs (0.17% ± 0.06%; 7/3,062 mCherry). Within the total population of targeted VGluT2^−^ VGaT^+^-expressing eYFP neurons (1,808 neurons, 3 mice; Figures 1D and 1G), we found that more than 90% expressed only VGluT2 mRNA (93.75% ± 2.28%; 1,722/1,808), rarely expressed VGaT mRNA alone (0.13% ± 0.13%; 2/1,808) or together with VGluT2 mRNA (1.09% ± 0.22%; 23/1,808), and a small number lacked VGluT2 and VGaT mRNAs (5.02% ± 2.34%; 61/1,808). Within the total population of targeted VGluT2^−^ VGaT^+^-expressing BFP neurons (755 neurons, 3 mice; Figures 1E and 1H), close to 98% expressed only VGaT mRNA (97.79% ± 0.35%; 733/755), none expressed VGluT2 mRNA alone, and infrequently had both VGluT2 and VGaT mRNA (2.21% ± 0.35%; 22/755). Taken together, these findings validate the selectivity of each of the INTRSECT2.0 viral vectors, providing the tools for the selective access of VGluT2^+^ VGaT^+^ (using AAV-C_ON_/F_ON_ vectors), VGluT2^+^ VGaT^−^ (using AAV-C_ON_/Flp_OFF_ vectors), and VGluT2^−^ VGaT^+^ neurons (using AAV-C_OFF_/F_ON_ vectors) within the VTA of *vglut2-Cre/vgat-Flp* mice.

**Figure 2.**
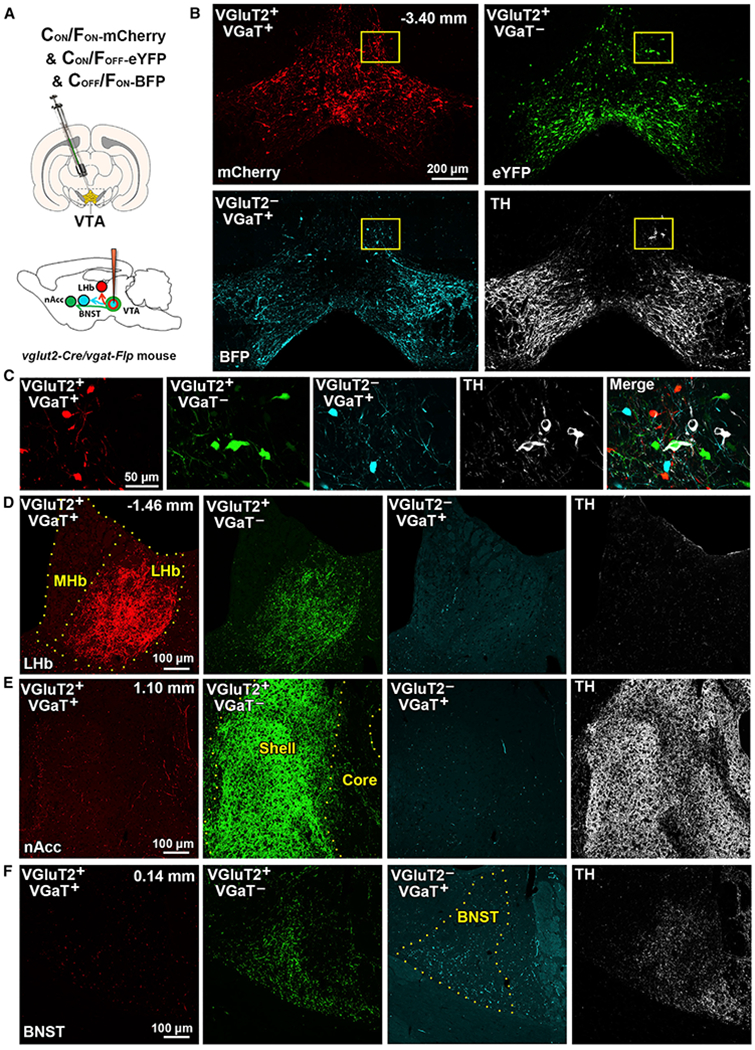
Projections of VTA VGluT2^+^ VGaT^+^, VGluT2^+^ VGaT^−^, and VGluT2^−^ VGaT^+^ Neurons (A) Schematic of injection of a cocktail of INTRSECT2.0 vectors (AAV-C_ON_/F_ON_-mCherry, AAV-C_ON_/F_OFF_-eYFP, and AAV-C_OFF_/F_ON_-BFP) into the VTA of vglut2-cre/*vgat-Flp* mice. (B) Detection of mCherry (targeting VGluT2^+^ VGaT^+^ neurons), eYFP (targeting VGluT2^+^ VGaT^−^ neurons), BFP (targeting VGluT2^−^ VGaT^+^ neurons), and TH immunoreactivity in VTA at low magnification. (C) At higher magnification, visualization of VTA mCherry, eYFP, BFP, or TH cell bodies. (D) Lateral habenula (LHb) with dense mCherry fibers and less dense eYFP or BFP fibers. (E) Nucleus accumbens shell (nAcc shell) with dense eYFP fibers and less dense mCherry or BFP fibers. (F) Bed nucleus of the stria terminalis (BNST) with BFP fibers and less dense mCherry or eYFP fibers.

Following the validation of INTRSECT2.0 recombination in each defined class of VTA neuron, we next injected into the VTA of *vglut2-Cre/vgat-Flp* mice a cocktail of the three INTRSECT2.0 vectors to simultaneously label VGluT2^+^ VGaT^+^ neurons (expressing mCherry), VGluT2^+^ VGaT^−^ neurons (expressing eYFP), and VGluT2^−^ VGaT^+^ neurons (expressing BFP; Figure 2). By confocal microscopy, we detected mCherry-, eYFP-, and BFP-expressing cell bodies within the VTA (Figures 2B and 2C) and fibers from these labeled neurons throughout the brain (Figures 2D–2F). Fluorescently labeled VTA cell types were observed throughout the VTA, but cell types showed differential proportions based on VTA subdivision (Figure S1). VGluT2^+^ VGaT^+^ and VGluT2^+^ VGaT^−^ neurons that lack TH co-expression were most often located in midline VTA subdivisions (rostral linear and interfascicular subdivisions). VGluT2^+^ VGaT^+^ neurons rarely co-expressed TH (0.28%; 4/1,410 VGluT2^+^ VGaT^+^ neurons). About one-quarter of VGluT2^+^ VGaT^−^ neurons co-expressed TH (25.83%; 897/3,473 VGluT2^+^ VGaT^−^), and these TH co-expressing VGluT2^+^ VGaT^−^ neurons were most often located in ventral VTA subdivisions (paranigral, interfascicular, and parainterfascicular subdivisions). VGluT2^−^ VGaT^+^ neurons rarely co-expressed TH (0.12%; 1/805 VGluT2^−^ VGaT^+^ neurons) and were most often located in lateral subdivisions (parabrachial pigmented subdivision).

The distribution and density of VTA cell-type-specific projections also differed. Although we observed mCherry fibers (from VGluT2^+^ VGaT^+^ neurons) in different brain structures, we detected the highest density in lateral habenula (LHb) (Figure 2D), followed by lateral hypothalamus (LH), less concentrated in central amygdala, dorsal striatum, lateral parabrachial nucleus, dorsal raphe, and nucleus accumbens (nAcc) (Figure 2E), and few in interstitial nucleus of the posterior limb of the anterior commissure and olfactory tubercle (Figures 2 and S2). We detected (1) higher density of VGluT2^+^ VGaT^+^ mCherry fibers than VGluT2^+^ VGaT^−^ eYFP or VGluT2^−^ VGaT^+^ BFP fibers in the LHb; (2) higher density of eYFP fibers (from VGluT2^+^ VGaT^−^ neurons) than VGluT2^−^ VGaT^+^ BFP fibers in the nAcc shell, olfactory tubercle, anterior cortical amygdaloid area, interstitial nucleus of the posterior limb of the anterior commissure, lateral preoptic area, central amygdala, and prefrontal cortex; and (3) a high density of VGluT2^−^ VGaT^+^ BFP fibers and VGluT2^+^ VGaT^−^ eYFP fibers in the bed nucleus of the stria terminalis (BNST) (Figure 2F), peduncular part of the lateral hypothalamus (PLH), and dorsal raphe (Figure S2).

**Figure 3.**
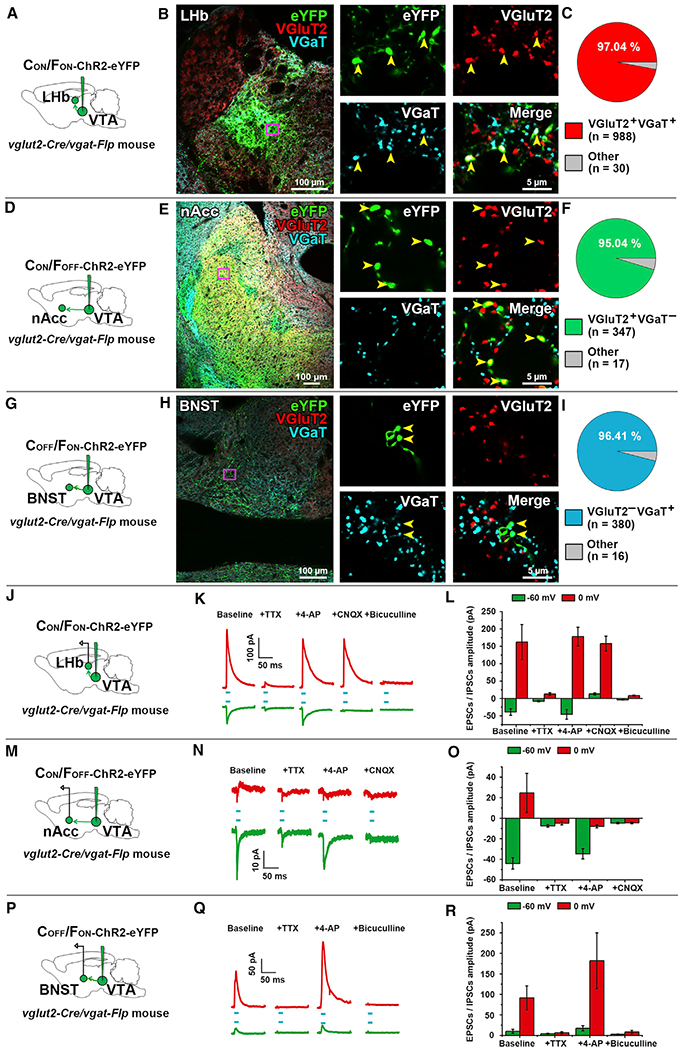
Vesicular Accumulation and Release of Glutamate or GABA by VTA VGluT2^+^ VGaT^+^, VGluT2^+^ VGaT^−^, and VGluT2^−^ VGaT^+^ Neurons (A) AAV-C_ON_/F_ON_-ChR2-eYFP injection in VTA of vglut2-cre/*vgat-Flp* mice. (B) LHb detection of eYFP, VGluT2, and VGaT, showing VGluT2 and VGaT expression in eYFP-positive axon terminals (arrows). (C) LHb co-expression of VGluT2 and VGaT in 97.04% of eYFP axon terminals (n = 3). (D) AAV-C_ON_/Flp_OFF_-ChR2-eYFP injection in VTA of vglut2-cre/*vgat-Flp* mice. (E) nAcc shell detection of eYFP, VGluT2, and VGaT, showing expression of VGluT2 in eYFP axon terminals, but not VGaT (arrows). (F) nAcc shell expression of VGluT2 without VGaT in 95.04% of eYFP axon terminals (n = 3). (G) AAV-C_OFF_/F_ON_-ChR2-eYFP injection in VTA of vglut2-cre/*vgat-Flp* mice YFP. (H) BNST detection of eYFP, VGluT2, and VGaT, showing expression of VGaT in eYFP axon terminals, but not VGluT2 (arrows). (I) BNST expression of VGaT without VGluT2 in 96.41% of eYFP axon terminals (n = 3). (J–R) Release of glutamate or GABA by VTA axon terminals. (J) AAV-C_ON_/F_ON_-ChR2-eYFP injection in VTA and LHb recordings. (K and L) LHb photostimulation (blue line) evoked excitatory postsynaptic currents (EPSCs) at −60 mV and inhibitory postsynaptic currents (IPSCs) at 0 mV. Currents were inhibited by TTX(at −60 mV: −38.9 ± 9.7 pA baseline and −7.9 ± 1.8 pA after TTX; at 0 mV: 162.5 ± 50.4 pA baseline and 12.6 ± 3.9 pA after TTX). Currents were restored by 4-AP (at −60mV: −38.9 ± 9.7 pA baseline and −45.8 ± 13.9 pA after 4-AP; at 0 mV: 62.5 ± 50.4 pA baseline and 178.2 ± 26.8 pA after 4 AP). EPSCs were blocked by CNQX (13.0 ± 2.9 pA) and IPSCs by bicuculline (8.2 ± 1.4 pA; −60 mV currents F_(4,29)_ = 13.09; ***p ^<^ 0.0001; repeated-measures ANOVA; Dunnett post hoc test n = 6 cells of 4 mice; 0 mV currents F_(4,29)_ = 13.89; ****p ^<^ 0.0001; repeated-measures ANOVA; Dunnett post hoc test n = 6 cells of 4 mice; *p ^<^ 0.05; **p ^<^ 0.01; ***p ^<^ 0.01). (M) AAV-C_ON_/F_OFF_-ChR2-eYFP injection in VTA and nAcc recordings. (N and O) nAcc photostimulation evoked EPSCs, which were inhibited by TTX (−44.1 ± 5.6 pA baseline and −7.4 ± 1.2 pA after TTX), restored by 4-AP (−44.1 ± 5.6 pA baseline and 34.7 ± 5.0 pA after 4-AP), and blocked by CNQX (4.7 ± 0.7 pA). (P) AAV-C_OFF_/F_ON_-ChR2-eYFP injection in VTA and BNST recordings. (Q and R) BNST photostimulation evoked IPSCs, which were inhibited by TTX (91.6 ± 29.0 pA baseline; 6.5 ± 2.5 pA after TTX), restored by 4-AP (182.0 ± 67.9 pA), and blocked by bicuculline (8.3 ± 4.0 pA). Data are mean ± SEM.

Given that the presence of fibers within a brain structure may correspond to fibers passing through or establishing synapses, we next determined whether VGluT2^+^ VGaT^+^ fibers established synapses in LHb, VGluT2^+^ VGaT^−^ neuron fibers established synapses in nAcc shell, or VGluT2^−^ VGaT^+^ neuron fibers established synapses in BNST. We first evaluated the extent to which VGluT2 protein or VGaT protein were present in axon terminals from VTA neurons innervating the LHb, nAcc shell, and BNST in *vglut2-Cre/vgat-Flp* mice with intra-VTA injections of INTRSECT vectors expressing channelrhodopsin (ChR2) tethered to eYFP (AAV-C_ON_/F_ON_-ChR2-eYFP, AAV-C_ON_/F_OFF_-ChR2-eYFP, or AAV-C_OFF_/F_ON_-ChR2-eYFP; Fenno et al., 2014; Figure 3). In the LHb of mice injected with AAV-C_ON_/F_ON_-ChR2-eYFP (to target VTA VGluT2^+^ VGaT^+^ neurons), we found that, within the total population of eYFP terminals (1,018 terminals), most of them co-expressed VGluT2 and VGaT (97.04% ± 0.39%; Figures 3A–3C). In the nAcc shell of mice injected with AAV-C_ON_/F_OFF_-ChR2-eYFP (to target VGluT2^+^ VGaT^−^ neurons), we found that, within the total population of eYFP terminals (364 terminals), most of them co-expressed VGluT2 (95.04% ± 0.80%; Figures 3D–3F), very few co-expressed VGaT (2.79% ± 0.46%,), and they rarely co-expressed both VGluT2 and VGaT (2.17% ± 0.40%). In the BNST of mice injected with AAV-C_OFF_/F_ON_-ChR2-eYFP (to target VGluT2^−^ VGaT^+^ neurons), we found that, within the total population of eYFP terminals (396 terminals), most of them co-expressed VGaT (96.41% ± 0.99%; Figures 3G–3I), none co-expressed VGluT2, and they infrequently co-expressed both VGluT2 and VGaT (3.59% ± 0.99%). These anatomical findings further support the cell-type-specific targeting of VTA neurons by INTRSECT vectors in *vglut2-Cre/vgat-Flp* mice.

Next, we prepared LHb slices to record the co-release of glutamate and GABA from VGluT2^+^ VGaT^+^ axon terminals from VTA-injected AAV-C_ON_/F_ON_-ChR2-eYFP mice (Figure 3J). By voltage-clamp recordings, we evaluated vesicular release of GABA (at 0 mV) or glutamate (at −60 mV) and found that LHb laser stimulation (5 ms) evoked outward currents at 0 mV and inward currents at −60 mV (Figure 3K). Both inward and outward currents were blocked by TTX (1 μM) and restored by 4-amino-pyradine (AP) (200 μM), demonstrating monosynaptic neurotransmitter release. Further, inward currents evoked at −60 mV were blocked by the AMPA-receptor antagonist CNQX (10 μM; without affecting the outward currents evoked at 0 mV), and the outward currents evoked at 0 mV were blocked by the GABA_A_-receptor antagonist bicuculline (10 μM; Figure 3L). These results further validate our intersectional approach for the specific targeting and manipulation of VTA VGluT2^+^ VGaT^+^ neurons and their axonal monosynaptic co-release of glutamate and GABA in LHb (Figures 3K and 3L).

We obtained nAcc slices to record the release of glutamate or GABA from VGluT2^+^ VGaT^−^ axon terminals from VTA-injected AAV-C_ON_/F_OFF_-ChR2-eYFP mice (Figure 3M) and found that nAcc laserstimulation evoked inward currents at −60 mV (blocked by CNQX) and small outward currents at 0 mV (Figures 3N and 3O). Both currents were blocked by TTX, but only inward currents were restored by 4-AP (Figures 3N and 3O), indicating that only the inward glutamatergic current was monosynaptic. These findings support the specific targeting and manipulation of VTA VGluT2^+^ VGaT^−^ neurons and their axonal monosynaptic release of glutamate in nAcc. We used BNST slices to record the release of glutamate or GABA from VGluT2^−^ VGaT^+^ fibers from VTA-injected AAV-C_OFF_/F_ON_-ChR2-eYFP mice (Figure 3P). We found that BNST laser stimulation evoked large-amplitude outward currents at 0 mV (blocked by bicuculline) and small-amplitude outward currents at −60 mV. Both currents were blocked by TTX, restored by 4-AP, and blocked by bicuculline (Figures 3Q and 3R), indicating monosynaptic inhibitory transmission. These findings further support the specific targeting of VTA VGluT2^−^ VGaT^+^ neurons and their axonal monosynaptic release of GABA within the BNST.

### VTA Cell-type-Specific Targeting of GCaMP6m

We next aimed to identify how each VTA cell type signals reward- and aversion-related motivated behaviors. First, we evaluated the cell-type-specific expression of the calcium indicator GCaMP6m in VGluT2^+^ VGaT^+^, VGluT2^+^ VGaT^−^, or VGluT2^−^ VGaT^+^ neurons (Figure S3).

To target GCaMP6m into VGluT2^+^ VGaT^+^ neurons, we injected AAV-C_ON_/F_ON_-GCaMP6m into the VTA of *vglut2-Cre/vgat-Flp* mice (n = 3; Figure S3C). By detection of VGluT2 and VGaT mRNAs, we determined that, within the total population of VTA GCaMP neurons (395 neurons), the majority (85.4% ± 2.4%; 332 neurons) co-expressed VGluT2 mRNA and VGaT mRNA, some expressed solely VGluT2 mRNA (9.7% ± 2.0%; 38 neurons), and few expressed solely VGaT mRNA (0.8% ± 0.5%; 3 neurons; Figure S3C). To target VGluT2^+^ VGaT^−^ neurons, we injected AAV-C_ON_/F_OFF_-GCaMP6m into the VTA of *vglut2-Cre/vgat-Flp* mice (n = 3; Figure S3D). We found that, within the total population of VTA GCaMP neurons (476 neurons), the majority (87.2% ± 0.1%; 415 neurons) expressed solely VGluT2 mRNA, none of them expressed solely VGaT mRNA, and they infrequently co-expressed VGluT2 and VGaT mRNA (1.7% ± 0.7%; 8 neurons; Figure S3D). To target GCaMP6m into VGluT2^−^ VGaT^+^ neurons, we injected the AAV-C_OFF_/F_ON_-GCaMP6m vector into the VTA of *vglut2-Cre/vgat-Flp* mice (n = 4; Figure S3E). We determined that, within the total population of VTA GCaMP neurons (904 neurons), most of them expressed solely VGaT mRNA (96.5% ± 0.01%; 872 neurons), none expressed solely VGluT2 mRNA, and they infrequently co-expressed VGluT2 and VGaT mRNAs (1.5% ± 0.01%; 18 neurons; Figure S3E). These findings support the selective and efficient targeting of GCaMP6m to VTA VGluT2^+^ VGaT^+^, VGluT2^+^ VGaT^−^, or VGluT2^−^ VGaT^+^ neurons.

### VTA Cell-type-Specific Responses to Reward and Reward-Related Cues

**Figure 4.**
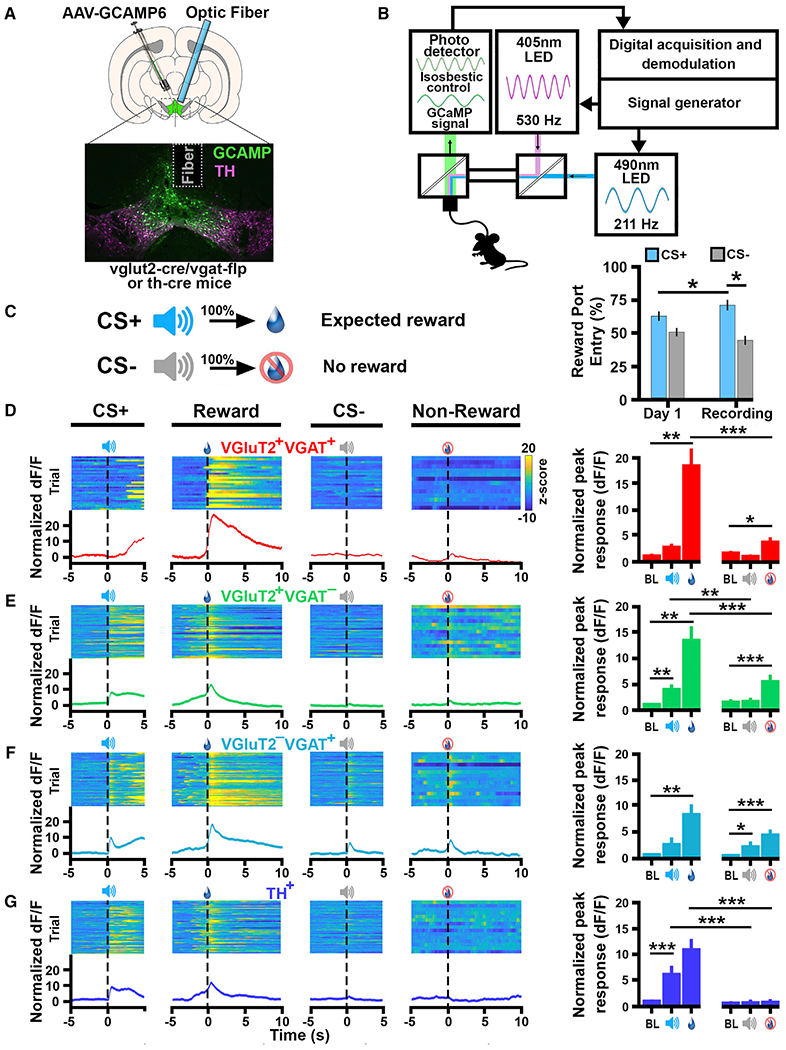
Response to Conditioned Reward by VTA VGluT2^+^ VGaT^+^, VGluT2^+^ VGaT^−^, VGluT2^−^ VGaT^+^, and TH^+^ Neurons (A) Schematic of injection of AAV-C_ON_/F_ON_-GCAMP6m,AAV-C_ON_/Flp_OFF_-GCAMP6m, or AAV-C_OFF_/F_ON_-GCAMP6m into VTA of vglut2-Cre/*vgat-Flp* mice or AAV-DIO-GCaMP6m into the VTA of *th-Cre* mice. optic fiber placement over GCAMP-positive cells in the VTA is shown. (B) Diagram of calcium imaging system. (C) Sucrose reward conditioning and testing procedures. Mice learned to respond more to the CS+ over training (p < 0.05) and to discriminate between CS+ and CS− (p < 0.0001; N = 6–10 per group; two-way ANOVA; session × cue type interaction; ANOVA; F_(1,28)_ = 5.69; p < 0.024). *p < 0.05 (D–G) Observed Ca^2+^ responses in the VTA during sucrose reward conditioning (left) and population average Ca^2+^ signals (right). BL, baseline. Data are mean ± SEM. Comparisons between event-related activity are as follows: *p < 0.05, **p < 0.01, and ***p < 0.0001. (D) VGluT2^+^ VGaT^+^ neurons showing lack of changes in Ca^2+^ signal by the CS+ or CS− but increased signal by sucrose delivery (p < 0.001). (E) VGluT2^+^ VGaT^−^ neuron showing increases in Ca^2+^ signal by the CS+ (p < 0.01) and by sucrose delivery (p < 0.001). (F) VGluT2^−^ VGaT^+^ neurons showing moderate increase in Ca^2+^ signal by the CS+ (p = 0.064), sucrose delivery (p < 0.01), and by the CS− (p = 0.041). (G) TH^+^ neurons showing increases in Ca2^+^ signal by the CS+ (p < 0.001) and by sucrose delivery (p < 0.01; N = 6–10 per group; mixed ANOVA; group × cue type × event interaction; F_(12,108)_ = 4.802; p < 0.0001; Sidak-correction for pos-hoc comparisons).

After establishing the cell-type-specific targeting of INTRSECT2.0 GCAMP6m viral vectors in *vglut2-Cre/vgat-Flp* mice, we recorded cell-type-specific calcium transients within VTA VGluT2^+^ VGaT^+^, VGluT2^+^ VGaT^−^, or VGluT2^−^ VGaT^+^ neurons (Figures 4A, 4B, and S4). We also recorded calcium transients within VTA TH^+^ neurons from *th-Cre* mice injected into the VTA with AAV-DIO-GCaMP6m. We trained all mice in a task in which a 10-s auditory cue (CS+ cue) signaled the delivery of sucrose (reward) and a different 10-s auditory cue signaled that sucrose would not be delivered (CS− cue; Figure 4B). Following 7–14 days of training, mice learned that the CS+ cue resulted in reward delivery and that the CS− cue did not, as indicated by an increase in the percent of trials in which mice entered the reward port following the CS+ cue (from 60.28% ± 03.25% on day 1 to 72.15% ± 03.36% on the recording day) and a decrease in the percent of trials in which mice entered the reward port following the CS− cue (48.80% ± 03.25% on day 1 to 42.46% ± 03.36% on recording days; Figure 4C). There were no significant differences in the number of training sessions between groups (Figure S5A). Once mice efficiently discriminated between the CS+ and CS− cues, we recorded VTA cell-type-specific Ca^2+^ activity by fiber photometry. There were no significant differences in percent of reward port entries following CS+ or CS− cues between groups (Figure S5B).

Within the VGluT2^+^ VGaT^+^ neurons, we did not detect changes in Ca^2+^ signal in response to the CS+ cue (Figures 4D and S6). In contrast, we found in VGluT2^+^ VGaT^−^ and TH^+^ neurons a significant increase in Ca^2+^ signaling in response to the CS+ cue when compared with either the pre-stimulus baseline (VGluT2^+^ VGaT^+^, p < 0.01; TH^+^, p < 0.001) or the CS− cue (Figures 4D and S6; VGluT2^+^ VGaT^−^, p < 0.01; TH^+^, p < 0.0001; Figures 4E, 4G, and S6). Although in VGluT2^−^ VGaT^+^ neurons we detected a moderate increase in Ca^2+^signal in response to the CS+ cue, this increase in activity was not statistically different from baseline (p = 0.064). In contrast, VGluT2^−^ VGaT^+^ neurons significantly increased Ca^2+^ signal over baseline in response to the CS− cue (p = 0.041; Figures 4F and S6). VGluT2^−^ VGaT^+^ neurons were the only cell type to significantly increase activity in response to the CS−.

In contrast to the heterogeneity in responses to the CS+ and CS− cues among the VTA VGluT2^+^ VGaT^+^, VGluT2^+^ VGaT^−^, VGluT2^−^ VGaT^+^, and TH^+^ neurons, we found that all these cell types increased their Ca^2+^ activity in response to reward delivery (Figures 4D–4G and S6; p < 0.01). However, the Ca^2+^ signal in VGluT2^+^ VGaT^+^, VGluT2^+^ VGaT^−^, and TH^+^ neurons was greater when mice sought reward (reward-port entries) during trials in which the reward was delivered as predicted (CS+ cue trials) than when mice sought reward (reward-port entries) during trials in which reward was predicted to be absent (CS− cue trials; all p < 0.001).

### VTA Cell-type-Specific Responses to Errors in the Prediction of Reward

**Figure 5.**
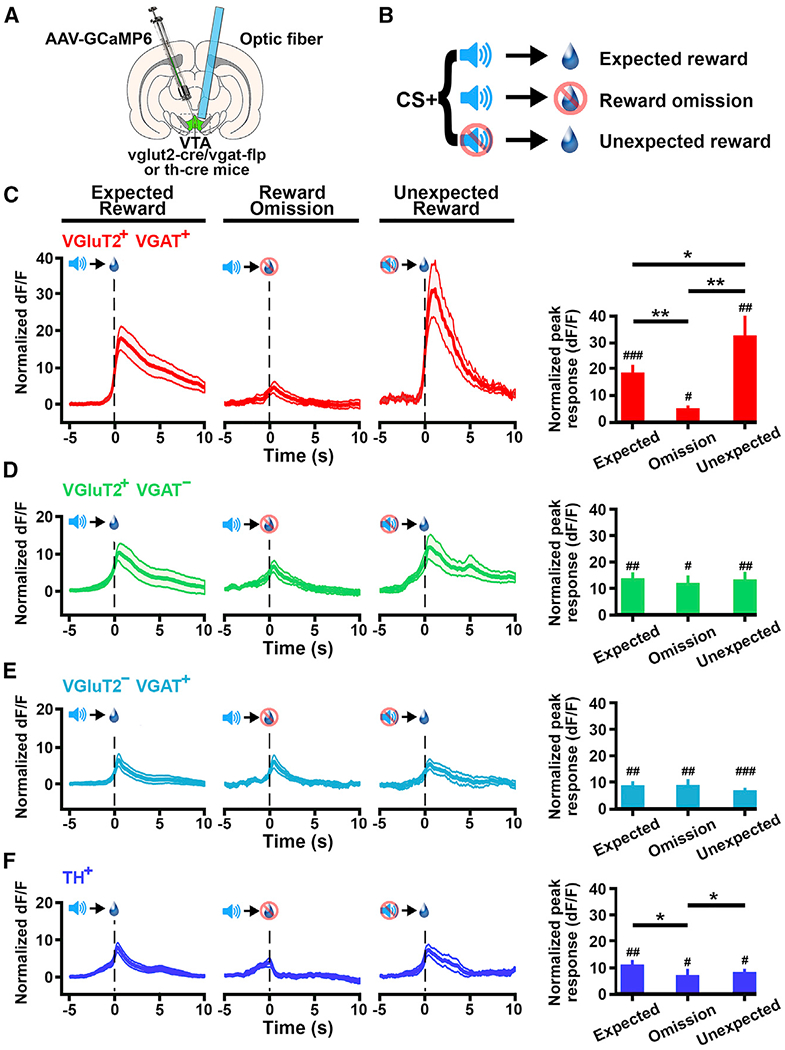
Response to Errors in the Prediction of Reward by VTA VGluT2^+^ VGaT^+^, VGluT2^+^ VGaT^−^, VGluT2^−^ VGaT^+^, and TH^+^ Neurons (A) Schematic of injection of AAV-C_ON_/F_ON_-GCAMP, AAV-C_ON_/Flp_OFF_-GCAMP6m, or AAV-C_OFF_/F_ON_-GCAMP6m into VTA of vglut2-Cre/*vgat-Flp* mice or AAV-DIO-GCaMP6m into the VTA of *th-Cre* mice. Optic fiber placement over GCAMP-positive cells in the VTA is shown. (B) Testing procedures in which VTA cell responses were recorded in response to CS+ and sucrose delivery (expected reward), CS+ and sucrose omission (reward-omission, error), or CS+ omission and sucrose delivery (unexpected reward). (C–F) Observed population responses during sucrose reward conditioning (left) and population average Ca^2+^ signals for each event (right). Data are mean ± SEM. Comparisons between event-related activity are as follows: *p < 0.05 and **p < 0.01. Comparisons to baseline activity are as follows: #p < 0.05; ##p < 0.01; and ###p < 0.001. (C) VGluT2^+^ VGaT^+^ neurons showing higher Ca^2+^ signal in response to expected sucrose versus its omission and even higher Ca^2+^ activity in response to unexpected sucrose (reward versus omission trials, p < 0.01; unexpected reward versus omission trials, p < 0.05). (D) VGluT2^+^ VGaT^−^ neurons showing similar increases in Ca^2+^ signals in response to expected sucrose, sucrose omission (p < 0.01), or unexpected sucrose (p < 0.01). (E) VGluT2^−^ VGaT^+^ neurons showing similar increase in Ca^2+^ signaling in response to expected sucrose, sucrose omission (p < 0.05), or unexpected sucrose (p < 0.001). (F) TH^+^ neurons higher Ca^2+^ signaling in response to expected or unexpected sucrose than when sucrose was omitted (p < 0.05).

Previous work has demonstrated that VTA neuronal activity of TH^+^ (Cohen et al., 2012), VGaT^+^ (Cohen et al., 2012), and VGluT2^+^ neurons (Root et al., 2018a) changes depending on whether a predicted reward is received or not. Thus, we compared the Ca^2+^ activity of VGluT2^+^ VGaT^+^, VGluT2^+^ VGaT^−^, VGluT2^−^ VGaT^+^, and TH^+^ neurons in response to the presentation of a cued reward, omission of the cued reward, or unexpected reward (Figures 5A and 5B).

We detected an increase in Ca^2+^ signal in VGluT2^+^ VGaT^+^ neurons in response to cued reward (p < 0.001), reward omission (p < 0.05), and unexpected reward (p < 0.01). These VGluT2^+^ VGaT^+^ neural responses were of different magnitudes (Figure 5C): the highest magnitude was in response to an unexpected reward (magnitude higher than in response to the cued expected reward; unexpected versus cued, p < 0.05; unexpected versus omission, p < 0.01), and the lowest was in response to reward omission (cued versus omission, p<0.01). In contrast, Ca^2+^ signal in VGluT2^+^ VGaT^−^ neurons was of similar magnitude in response to cued reward (p <0.01), reward omission (p <0.05), or unexpected reward (p < 0.01; Figure 5D). In common with VGluT2^+^ VGaT^−^ neurons, Ca^2+^ signals in VGluT2^−^ VGaT^+^ neurons were also of similar magnitude in response to cued reward (p < 0.01), reward omission (p < 0.05), or unexpected reward (p < 0.001; Figure 5E). Although we also observed an increase in Ca^2+^ signal in TH^+^ neurons in response to reward delivery (p < 0.01), its omission (p < 0.05), or its unexpected delivery (p < 0.01), there was similar magnitude in the responses to cued and unexpected reward, but the response to reward omission was significantly lower than those in response to cued reward (p < 0.05) or unexpected reward (p < 0.05; Figure 5F).

### VTA Cell-type-Specific Responses to Aversive Stimuli and Aversion-Predicting Cues

**Figure 6.**
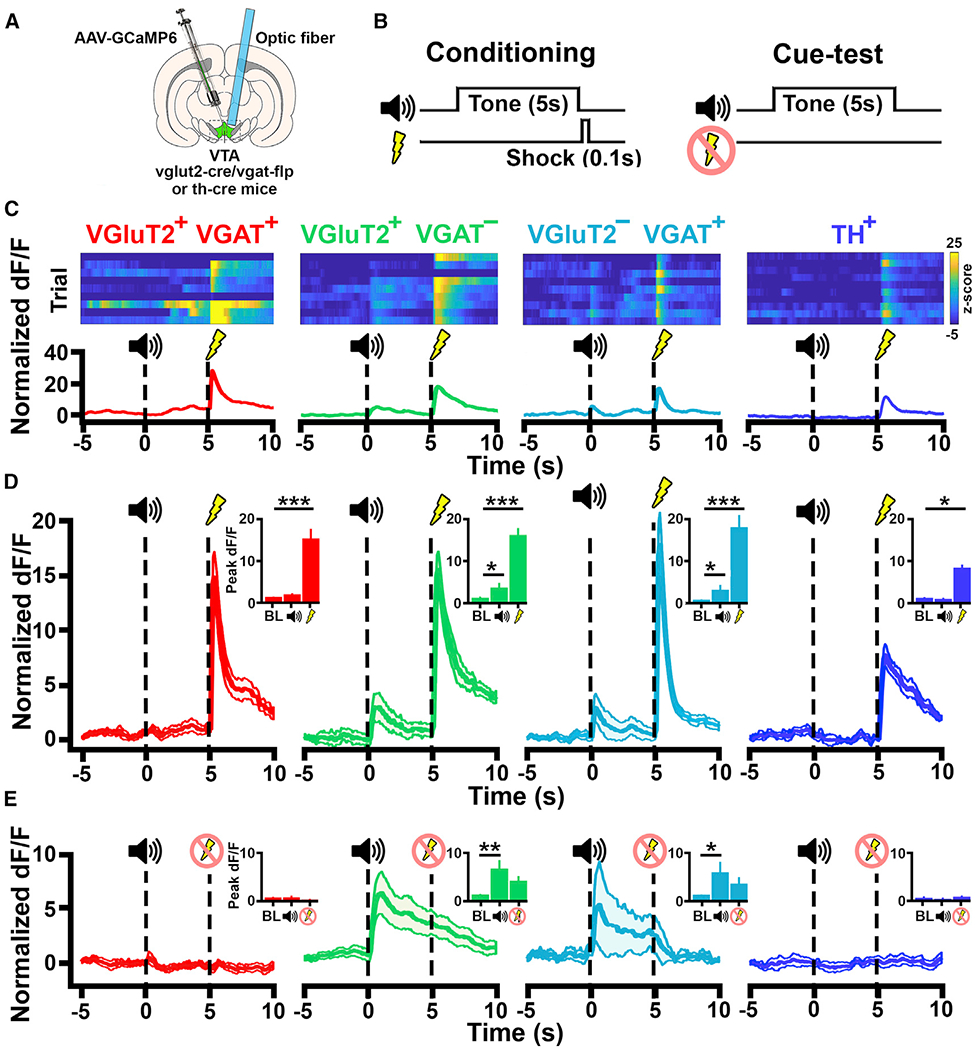
Response to Aversive Stimuli by VTA VGluT2^+^ VGaT^+^, VGluT2^+^ VGaT^−^, VGluT2^−^ VGaT^+^, and TH^+^ Neurons (A) Schematic of injection of AAV-C_ON_/F_ON_-GCAMP, AAV-C_ON_/Flp_OFF_-GCAMP6m, or AAV-C_OFF_/F_ON_-GCAMP6m into VTA of vglut2-Cre/vgat-Flp mice or AAV-DIO-GCaMP6m into the VTA of *th-Cre* mice. Optic fiber placement over GCAMP-positive cells in the VTA is shown. (B) Schematic of fear-conditioning training and cue-test sessions. (C) Heatmaps of Ca^2+^ signal over successive trials of fear-conditioning (during cue and footshock presentations). (D) Cell population responses to cue and shock in conditioning training showing increases in Ca^2+^signal in VGluT2^+^ VGaT^+^, VGluT2^+^ VGaT^−^, VGluT2^−^ VGaT^+^, and TH^+^ neurons in response to footshock (VGluT2^+^ VGaT^+^, p < 0.001; VGluT2^+^ VGaT^−^, p< 0.001; VGluT2^−^ VGaT^+^, p < 0.001; TH^+^, p< 0.05) and in response to the cue predicting the shock for VGluT2^+^ VGaT^−^ (p < 0.05) and VGluT2^−^ VGaT^+^ cell populations (p < 0.05). Data are mean ± SEM. Comparisons between event-related activity are as follows: *p < 0.05, **p < 0.01, ***p < 0.001. (E) Cell population average Ca^2+^ signals show increases in Ca^2+^ during the cue test for VGluT2^+^ VGaT^−^ (p< 0.05) and VGluT2^−^ VGaT^+^ neurons (p< 0.05), demonstrating a memory for the shock-paired cue. Data are mean ± SEM. Comparisons between event-related activity are as follows: *p < 0.05, **p < 0.01, ***p < 0.001.

Following training in the reward task, we recorded VTA neuronal activity in the same mice in a conditioning session in which a CS+ cue signaled the delivery of a brief footshock and tested responses to the CS+ cue 24 h following the conditioning session in the absence of footshock (Figures 6A, 6B, and S7). During shock-conditioning sessions, we observed that all mice (VGluT2^+^ VGaT^+^, VGluT2^+^ VGaT^−^,VGluT2^−^ VGaT^+^, and TH^+^) exhibited rapid cue learning. We also detected increases in freezing behavior following CS+ presentation during the cue test, as compared to a group of mice that received only tone CS+ cue presentations without footshock delivery (tone-only control group; F(4,28) = 10.275, p< 0.001; post hoc Sidak-adjusted all p <0.01; Figure S7B), demonstrating the behavioral expression of fear following the presentation of the footshock-predicting CS+. In all VTA cell types (VGluT2^+^ VGaT^+^, VGluT2^+^ VGaT^−^, VGluT2^−^ VGaT^+^, and TH^+^), we observed increases in Ca^2+^ signal in response to footshock during shock conditioning sessions, as compared to baseline (VGluT2^+^ VGaT^−^, p < 0.001; VGluT2^−^ VGaT^+^, p < 0.001; VGluT2^+^ VGaT^+^, p < 0.001; TH, p < 0.05; Figure 6C).

In common with responses in the reward-learning task, we did not observe increases in Ca^2+^ signal in VGluT2^+^ VGaT^+^ neurons in response to the CS+ during either the shock conditioning session or the subsequent cue test (Figures 6C–6E). Also in common with responses found in the reward learning task, we observed increases in Ca^2+^ signal in VGluT2^+^ VGaT^−^ neurons in response to the CS+ during both the shock-conditioning session (p < 0.05) and the cue test (p < 0.01; Figures 6C–6E). In contrast with the reward-learning task where Ca^2+^ signals in VGluT2^−^ VGaT^+^ neurons did not significantly change activity in response to the reward-predicting CS+ cue, we observed in these neurons significantly increased Ca^2+^ signal in response to the CS+ cue, predicting shock during both the conditioning session and the cue test (Figures 6C–6E). We did not detect increases in Ca^2+^ signal in TH^+^ neurons in response to the shock-paired CS+ cue.

## DISCUSSION

Decades of research have demonstrated that VTA dopamine neurons participate in different aspects of motivated behavior (Blackburn et al., 1992; Bromberg-Martin et al., 2010; Ikemoto and Panksepp, 1999; Salamone et al., 2005; Wise and Rompre, 1989), and recent studies have shown that VTA GABA neurons (Tan et al., 2012; van Zessen et al., 2012) and VTA glutamate neurons also participate in motivated behavior (Bimpisidis et al., 2019; Lammel et al., 2015; Root et al., 2014a). Moreover, VTA cellular recordings of GABA neurons in *vgat-Cre* mice (Cohen et al., 2012) and glutamate neurons in *vglut2-Cre* mice (Root et al., 2018a) have shown that some of these neurons may signal reward, aversion, or cues that predict them. Given that the VTA has glutamate-GABA co-transmitting neurons (VGluT2^+^ VGaT^+^ neurons), glutamate-transmitting neurons (VGluT2^+^ VGaT^−^), and GABA-transmitting neurons (VGluT2^−^ VGaT^+^; Root et al., 2014b, 2018b), it is possible that properties previously ascribed to VTA GABA (using *vgat-Cre* mice) or glutamate (using *vglut2-Cre* mice) neurons may correspond to combinatorial glutamate-GABA co-transmitting neurons.

By photometric recordings of VTA genetically defined neurons, we demonstrated that glutamate-GABA co-transmitting neurons are functionally distinct from both glutamate-transmitting and GABA-transmitting neurons. We found that, although VTA glutamate-GABA co-transmitting neurons did not increase their activity in response to cues that predicted reward, cues that predicted reward absence, or cues that predicted aversive outcomes, they did increase their activity in response to both rewarding and aversive outcomes. In contrast, we found that VTA glutamate-transmitting neurons increased their activity in response to cues predicting reward or aversion and in response to rewarding and aversive outcomes, but these neurons were not activated by cues predicting the absence of reward. Moreover, we found that VTA GABA-transmitting neurons also had a unique neuronal activity profile, as evidenced by their increased activity in response to cues predicting the absence of reward, cues predicting the delivery of an aversive stimulus, and in response to both rewarding and aversive outcomes. Furthermore, we found that the neuronal activity of glutamate-GABA co-transmitting neurons, but not glutamate-transmitting or GABA-transmitting neurons, was modulated by errors in the predicted delivery of reward. The increased activity of VTA glutamate-GABA co-transmitting neurons in response to predicted reward delivery was lowered by reward omission and elevated by an unexpected reward. In contrast, we observed that VTA glutamate-transmitting and GABA-transmitting neurons did not show differences in activity between predicted, unpredicted, or omitted reward conditions. In addition, we determined that the activity of VTA TH neurons was biased toward rewarding conditions, as these neurons increased their activity in response to cues predicting reward and in response to reward, showed decreased activity following reward omission, and had no response to cues signaling the absence of reward or to cues predicting the delivery of footshock. Though TH neurons were activated by footshock, they showed the lowest footshock-related activity among all types of recorded neurons. Collectively, these findings demonstrate the distinct signaling of genetically defined VTA neurons in specific aspects of motivated behavior.

### Selective Targeting of VTA Neurons

To access the different types of VTA neurons, we used newly developed INTRSECT2.0 viral vectors (Fenno et al., 2020), which under the regulation of Cre and Flp recombinases (together or separately) encoded fluorescent reporter molecules (mCherry, eYFP, or BFP), GCaMP, or ChR2. We used these different viral vectors separately or as a cocktail for VTA cell-type-specific transduction in *vglut2-Cre/vgat-Flp* mice and validated the VTA cell-specific targeting of the viral vectors by a series of anatomical evaluations and *ex vivo* recordings.

By histological analysis, we demonstrated that most of the VTA transduced neurons with intersectional C_ON_/F_ON_ viral vectors in *vglut2-Cre/vgat-Flp* mice, for targeting glutamate-GABA co-transmitting neurons, co-expressed VGluT2 mRNA and VGaT mRNA. By confocal microscopy detection of fluorescent reporter proteins and immunodetection of endogenous VGluT2 protein and VGaT protein, we confirmed that axon terminals from C_ON_/F_ON_-transduced VTA neurons co-expressed VGluT2 and VGaT proteins within the LHb. Moreover, by LHb *ex vivo* electrophysiological recordings, we demonstrated that axon terminals from C_ON_/F_ON_-transduced VTA neurons drove both excitatory and inhibitory postsynaptic currents in LHb neurons, indicating the co-transmission of glutamate and GABA from the VTA-transduced neurons.

We showed that most of the VTA-transduced neurons with subtractive C_ON_/F_OFF_ viral vectors in *vglut2-Cre/vgat-Flp* mice, for targeting glutamate-transmitting neurons, expressed VGluT2 mRNA without VGaT mRNA; expressed VGluT2 proteins, but not VGaT proteins, in their axon terminals within the nAcc; and released glutamate without GABA in the nAcc. We also demonstrated that most of the VTA-transduced neurons with subtractive C_0ff_/F_0n_ viral vectors in *vglut2-Cre/vgat-Flp* mice, for targeting GABA-transmitting neurons, expressed VGaT mRNA without VGluT2 mRNA; expressed VGaT proteins, but notVGluT2 proteins, in their axon terminals within the BNST; and released GABA without glutamate in the BNST. Furthermore, the spatial distribution of each class of VTA-transfected neurons matched our previous anatomical findings in wild-type rats and mice, showing that both VGluT2^+^ VGaT^+^ and VGluT2^+^ VGaT^−^ neurons are concentrated in the midline VTA and VGluT2^−^ VGaT^+^ neurons are concentrated in the lateral and posterior VTA (Morales and Root, 2014; Root et al., 2014b, 2018b; Yamaguchi et al., 2007, 2011, 2015). Together, these results provide validation for the INTRSECT2.0 viral strategies and confirm that our application here resulted in the selective targeting of genetically defined VTA neurons that release both glutamate and GABA, release glutamate without GABA, or release GABA without glutamate. We also detected a subpopulation of VTA VGluT2^+^ VGaT^−^ neurons co-expressing TH, indicating that VGluT2^+^ VGaT^−^ neurons include neurons that release glutamate without GABA or dopamine and another subpopulation that may release glutamate and dopamine, but not GABA. A third recombinase under the control of the TH promoter would be necessary to gain genetic access to these two cell types. Although recently developed INTRSECT2.0 vectors allow neuronal access based on three genetic characteristics (Fenno et al., 2020), further research will be necessary to genetically dissect VTA neurons based on more than two genetic characteristics.

Using INTRSECT2.0 vectors to identify the efferent targets of each VTA cell type, we found that VTA glutamate and GABA cell types show differential densities in their projection targets. LHb receives most VTA inputs from glutamate-GABA neurons, accumbens shell receives most VTA inputs from glutamate neurons, and dorsal raphe receives most VTA input from GABA neurons. All cell types showed additional targets, but glutamate neurons had the most widespread dense targets, including olfactory tubercle, interstitial nucleus of the posterior limb of the anterior commissure, lateral preoptic area, central amygdala, anterior cortical amygdaloid area, and prefrontal cortex. Together, these results refine the known cell-type-specific targets of VTA glutamate and GABA neurons (Li et al., 2019; Qi et al., 2016; Root et al., 2014b; Taylor et al., 2014; Yamaguchi et al., 2011).

### VTA Neuronal Signaling of Reward- and Aversion-Related Outcomes and Predictors

Genetically defined subpopulations of VTA neurons showed distinct patterns of neuronal activity in response to reward, to aversion, to learned cues predicting rewarding and aversive stimuli, and to errors in the prediction of reward (Table 1). Prior studies have shown that subsets of optogenetically identified VTA VGaT neurons (Cohen et al., 2012; Eshel et al., 2015) or VGluT2 (Root et al., 2018a) neurons increase their firing rates in response to cues predicting reward, as well as cues that predict the absence of reward. We determined that VTA glutamate-transmitting neurons increased their activity in response to cues predicting reward, but not in response to cues predicting the absence of reward. In contrast, we found that VTA GABA-transmitting neurons increased their activity in response to cues predicting the absence of reward and, to a lesser extent, in response to cues predicting reward. Further, we identified in glutamate-GABA co-transmitting neuron two signaling features that distinguished them from the other classes of VTA recorded cell types: first, their signaling of rewarding or aversive outcomes without any change in activity in response to cues predicting reward, cues predicting the absence of reward, or cues predicting footshock, and second, their selective modulation by errors in the receipt of a predicted reward.

By photometric recordings, we found that, although the activity of both glutamate-transmitting and GABA-transmitting neurons increased in response to the receipt of the predicted reward, the magnitude of the responses was similar whether the predicted reward was delivered or omitted. These findings suggest that VTA glutamate-transmitting and GABA-transmitting neurons signal reward expectation. In contrast, we found that the activity of glutamate-GABA co-transmitting neurons was higher in response to reward delivery when compared with reward omission, and the reward response was the highest following an unexpected reward (compared with an expected reward). Thus, we suggest that the reward-related responses by VTA glutamate-GABA neurons reflects aspects of expectation different than those involving VTA glutamate-transmitting or GABA-transmitting neurons. We infer that VTA glutamate-GABA neurons signal violations of reward expectation, whereas both glutamate-transmitting and GABA-transmitting neurons signal reward expectation itself. It was recently proposed that glutamate-dopamine co-releasing neurons have a role in “behavioral switching” (Mingote et al., 2019), suggesting that both glutamate-GABA and glutamate-dopamine co-releasing neurons may influence behavior under changing conditions.

We also found that VTA dopamine neurons, in common with glutamate-GABA co-transmitting neurons, showed higher activity in response to reward delivery than in response to reward omission. However, in contrast to the lack of response to cues predicting reward by glutamate-GABA co-transmitting neurons, we confirmed that the activity of dopamine neurons increased in response to a cue predicting reward. These findings indicate that dopamine neurons differ from glutamate-GABA co-transmitting neurons in their capacity to signal predicted rewards, but not their capacity to signal the presence of reward versus its absence. One hypothesis regarding the role of VTA GABA neurons during reward expectation suggests that the activation of GABA neurons serves to inhibit VTA dopamine neurons at the time of the expected reward (Eshel et al., 2015). From our findings, we infer that the VTA GABA neurons proposed to inhibit the firing of neighboring dopamine neurons are likely to belong to the group of GABA-transmitting neurons given that these neurons, but not glutamate-GABA co-transmitting neurons, signal reward expectation.

Prior VTA electrophysiological recordings of phototagged VGaT (Cohen et al., 2012) and phototagged VGluT2 neurons (Root et al., 2018a) have shown that these neurons increase their activity in response to facial airpuffs and that the activity of VGaT neurons increases by cues predicting this aversive stimulus (Cohen et al., 2012). We further extend these observations by showing that, in response to footshock, there were increases in neuronal activity in glutamate-GABA co-transmitting, glutamate-transmitting, GABA-transmitting, and dopamine neurons. Moreover, we found that the neuronal activity of both glutamate-transmitting and GABA-transmitting neurons increased in response to cues predicting footshock. In contrast, we observed that both glutamate-GABA co-transmitting and dopamine neurons did not increase their activity in response to cues predicting footshock. These findings further indicate that a defining feature of VTA glutamate-GABA neurons is their signaling of rewarding or aversive outcomes together with a lack of responsiveness to learned cues that predict these outcomes.

Although findings from extracellular single-unit recordings have demonstrated decreases in neuronal activity in a subset of VTA dopamine neurons following aversive stimulation (Cohen et al., 2012), we did not observe decreased calcium signaling by VTA dopamine neurons in response to footshock. The lack of observed decreased calcium signaling may be explained by the fact that a prolonged footshock stimulation is required for the detection of decreased calcium signaling by VTA dopamine neurons in response to footshock (Kim et al., 2016). Furthermore, differential responses to aversive stimulation by VTA dopamine neurons may be explained by their location. Although we recorded calcium signaling in VTA dopamine neurons located near the medial VTA, extracellular single-unit recordings of dopamine neurons have predominately been located in lateral VTA (Cohen et al., 2012).

In summary, by using *vglut2-Cre/vgat-Flp* mice and INTRSECT2.0 viral vectors, we identified genetically defined VTA neuronal circuits and signaling of different aspects of motivated behavior. From this approach, we found that VTA glutamate-GABA co-transmitting, glutamate-transmitting, and GABA-transmitting neurons have differential projection densities as well as unique neuronal activity patterns in response to rewarding and aversive stimuli and their learned predictors. We concluded that, although glutamate-GABA co-transmitting neurons signal rewarding and aversive outcomes and their reward-related signals are modulated by errors in their predicted receipt or omission, they lack signaling related to learned cues predicting rewarding or aversive outcomes. Further, whereas glutamate-transmitting neurons signal cues predicting rewarding or aversive outcomes, the GABA-transmitting neuron signals cues predicting the absence of reward as well as cues predicting aversive outcomes.

## STAR★METHODS

### RESOURCE AVAILABILITY

#### Lead Contact

Further information and requests for resources and reagents should be directed to and will be fulfilled by the Lead Contact, Dr. Marisela Morales (MMORALES@intra.nida.nih.gov)

#### Materials Availability

This study did not generate new unique reagents.

#### Data and Code Availability

All data generated and codes created during the current study are available from the lead author upon reasonable request.

### EXPERIMENTAL MODEL AND SUBJECT DETAILS

Both male and female mice were used in the study (20-30 g). The mice used were VGluT2-IRES::Cre mice (JAX # 016963 and VGaT::FlpO mice (Obtained from The Allen Institute; (Daigle et al., 2018) Jax # 031331) that were crossed to produce a VGluT2-IRES::Cre x VGaT::FlpO mouse. Animals were housed in temperature- and humidity-controlled facilities under a 12 h light/dark cycle with dawn at 0700 h and *ad libitum* chow and water prior to the start of experimental procedures. Mice were 2-3 months of age at the start of the experiment. Experiments were conducted in accordance with the USPHP *Guide for the Care and Use of Laboratory Animals* and approved by the Animal Care and Use Committee of the National Institute on Drug Abuse Intramural Research Program.

### METHOD DETAILS

#### Surgery and virus injections

Mice were anesthetized with isoflurane (1%–4% induction;1% maintenance) and secured to a stereotaxic frame. After exposing the top of the skull, the mouse’s head was leveled to ensure the dorsoventral distances between bregma and lambda were within 100 mm of one another. Viruses were injected into the VTA (0.3 μl; AP: −3.2 to −3.4, ML: ± 0.0 to 0.3, DV: −4.3 to −4.4). Injections were made using a Micro4 controller and UltraMicroPump along with 10 ml Nanofil syringes equipped with 35-gauge needles (WPI Inc., Sarasota, FL). Syringes were left in place for 7-10 min following injections to minimize diffusion. For fiber photometry calcium imaging experiments, a 400 μm core optic fiber (Doric Lenses) embedded in a 2.5 mm ferrule was implanted over the VTA (AP: −3.2 to −3.4, ML: ± 0.0 to 0.3, DV: −4.3 to −4.4) and secured to the skull using #000 screws (Fasteners and Metal products Corp; #000-120X1/16) and dental cement. Following surgery, mice recovered on a warm heating pad before being transferred back to the vivarium. Mice remained in the colony to allow for recovery and virus expression for 3-5 weeks prior to the start of behavioral testing.

#### Histological verification of optic fibers

Based on our recent findings on VTA distribution of VGluT2^+^ VGaT^+^, VGluT2^+^ VGaT^−^, and VGluT2^−^ VGaT^+^ VGluT2-VGaT, VGluT2-only and VGaT-only neurons (Root et al., 2018b), we stereotaxically targeted optic fibers toward the medial aspects of the VTA to record VGluT2^+^ VGaT^+^ VGluT2-VGaT neurons (328.99 ± 42.41 μm relative to the midline) or VGluT2^+^ VGaT^−^ VGluT2-only (383.67 ± 70.63 μm relative to the midline), and slightly more lateral (453.54 ± 49.62 μm relative to the midline) to record VGluT2^−^ VGaT^+^ VGaT-only neurons. Given the VTA lateromedial heterogeneity of TH neurons (Li et al., 2013), we aimed to record lateral TH^+^-neurons (286.36 ± 68.68 μm relative to the midline; Figures S4A and S4B). However, we found that optic fibers were equivalent distances from the dorsal border of the VTA for the four classes of recorded neurons (122.06 ± 54.59 μm for VGluT2^+^ VGaT^+^, 123.86 ± 24.83 μm for VGluT2^+^ VGaT^−^ VGluT2-only, 106.36 ± 43.78 μm for VGluT2^−^ VGaT^+^ VGaT-only; and 36.54 ± 23.41 μm for TH^+^; F(3,32) = 0.869, p = 0.469, N.S.; Figure S4C). Further, photometry fibers were all at equivalent distances from the nearest identified GCAMP-positive cell (VGluT2^+^ VGaT^+^: 145.19 ± 31.38 μm; for VGluT2-VGaT VGaT-only VGluT2^−^ VGaT^+^: 118.88 ± 29.27 μm; VGluT2^+^ VGaT^−^: 128.99 ± 22.16 μm; for VGluT2-only VGluT2^+^ VGaT^−^: 128.99 ± 22.16 μm; and TH^+^: 107.17 ± 13.07 μm for TH^+^ 107.17 ± 13.07 μm; F(3,32) = 0.311, p = 0.817, N.S.; Figure S4D), and all of them within the distance necessary to effectively record GCaMP signals using a 400 μm 0.48NA fiber (Pisanello et al., 2018).

#### *In situ* hybridization

##### Tissue preparation

Three to five weeks following virus injections, mice were anesthetized with chloral hydrate (0.5 ml/kg) and perfused transcardially with 4% (w/v) paraformaldehyde (PF) in 0.1 M phosphate buffer (PB), pH 7.3. Brains were left in 4% PF for 2 h and transferred to 18% sucrose in PB overnight at 4°C. For *in situ* hybridization experiments coronal serial cryosections (16 μm) were prepared.

##### Phenotyping of VGluT2^+^ VGAT^+^, VGluT2^+^ VGAT^−^ and VGluT2^−^ VGAT^+^ neurons by in situ hybridization

Sections for the detection of both VGluT2 and VGaT in mice injected with AAV8-ef1α-CreON/FlpON-mCherry were first examined using a radioactive and chromogenic *in situ* hybridization. Sections were hybridized for 16 h at 55°C with [^35^S]- and [^33^P]-labeled (107 c.p.m./ml) rat single-stranded antisense probes for *VGluT2* (nucleotides 317^−^2357; GenBank accession code NM_053427) together with single-stranded rat mouse digoxigenin (DIG)-labeled *GAD65* and *GAD67* probes (nucleotides 1^−^1758, accession code NM_012563; nucleotides 1–1782, accession code NM_017007), or mouse *VGaT* (nucleotides 1-2814, GenBank accession code BC052020). To develop the DIG signal, sections were incubated with an alkaline phosphatase-conjugated antibody against DIG (Roche Applied Science; Indianapolis, IN) for 3 h at room temperature (RT); alkaline phosphatase reaction was developed with nitro bluetetrazolium and 5-bromo-4-chloro-3-indolyl phosphate (Life Technologies; Gaithersburg, MD), yielding a purple reaction product. Sections were then photographed under bright-field illumination. Sections were mounted on coated slides, dipped in Ilford K.5 nuclear tract emulsion (Polysciences; 1:1 dilution in double-distilled water), and exposed in the dark at 4°C for 3–4 weeks before development.

The remaining *in situ* hybridization experiments detected transcripts encoding of VGluT2 or VGaT using RNAscope. Sections were mounted onto Fisher SuperFrost slides and dried overnight at 60°C. RNAscope *in situ* hybridization was performed according to the manufacturer’s instructions. Briefly, sections were treated with heat and protease digestion followed by hybridization with a mixture containing target probes to mouse GAD (GAD65 and GAD67), VGluT2, and VGaT. Additional sections were hybridized with the bacterial gene DapB as a negative control, which did not exhibit fluorescent labeling. GAD was detected by Atto 647, VGluT2 by Atto 550, and VGaT by Alexa 488.

##### Data analysis of in situ hybridization studies

Radioactive *in situ* hybridization sections were viewed, analyzed and photographed with bright-field or epiluminescence microscopy using an Olympus VS120 microscope. Neurons were observed in each traced structure at high power (20 ×) and marked electronically. VGluT2, GAD, and VGaT labeled material was analyzed using epiluminescence to increase the contrast of silver grains identically to the manner reported previously by our laboratory (Root et al., 2014b; Yamaguchi et al., 2011). For all radioactive *in situ* hybridization *(VGluT2* mRNA), a cell was considered to express transcripts when its soma contained concentric aggregates of silver grains above background level. Radioactive *in situ* hybridization (silver grains) and nonradioactive *in situ* hybridization double-labeled material was analyzed by the following procedure: (i) silver grains corresponding to *VGluT2* mRNA expression were focused under epiluminescence microscopy, (ii) the path of epiluminescence light was blocked without changing the focus, and (iii) bright-field light was used to determine whether a purple neuron, expressing GAD or VGaT in focus, contained the aggregates of silver grains seen under epiluminescence.

RNAscope *in situ* hybridization sections were viewed, analyzed, and photographed with an Olympus FV1000 confocal microscope or a Keyence BZ-X800 Microscope. Negative control hybridizations showed negligible fluorophore expression. Neurons were counted when the stained cell was at least 5 μm in diameter. Pictures were adjusted to match contrast and brightness by using Adobe Photoshop (Adobe Systems). The number of mice (N = 3-4/group) analyzed was based on previous studies in our lab using radioactive detection of *VGluT2* mRNA from rat VTA neurons (Root et al., 2014b; Yamaguchi et al., 2011).

#### Cell body labeling and anterograde tracing

VGluT2-Cre/VGaT-Flp mice (6-12 weeks, 20-30 g) were anesthetized with 1%–5% isoflurane. Mice were injected in VTA with a mixture of three INTRSECT2.0 vectors: AAV8-ef1a-C_ON_/F_ON_-mCherry, AAV8-ef1α-C_ON_/F_OFF_-eYFP, AAV8-ef1α-C_OFF_/F_ON_-BFP (−3.3AP, 0ML, −4.3DV, 500 nL, 100 nL/min). Syringes were left in place for 10 min following injections to minimize diffusion. Following surgery, mice were housed in groups of 1-5 with a 12h:12h light/dark cycle. Six weeks following virus injection, mice were anesthetized with chloral hydrate (0.5 ml/kg) and perfused transcardially with 4% (w/v) paraformaldehyde (PF) in 0.1 M phosphate buffer (PB), pH 7.3. Brains were left in 4% PF for 2 h and transferred to 18% sucrose in PB overnight at 4°C. Brains were coronally sectioned (30 μm) and stored in six sequential tubes. One tube was incubated with blocking solution (4% bovine serum albumin (BSA) in PB supplemented with 0.3% Triton X-100) for 1 h. Sections were then incubated with sheep anti-TH (1:1000) overnight at 4C. Sections were washed and incubated for 2 h at room temperature with anti-sheep Alexa647 (1:100), coverslipped and confocally imaged. Brain regions for imaging were chosen based on targets of VTA glutamate and GABA-releasing neurons (Taylor et al., 2014). Confocal images (20X, Olympus FV1000) were acquired using identical pinhole, gain, and laser settings for all brain regions. Fluorescently-labeled VTA cell bodies that did or did not co-express TH-immunoreactivity were counted in Adobe Photoshop at bregma −2.92, −3.08, −3.16, −3.28, −3.40, −3.52, −3.64, −8.90, −3.88, −4.04, and −4.16 (mm from bregma) totalling 11 sections each from three mice. Neurons were counted individually by three scorers and the cell was counted if the soma was at least 5 mm in diameter. VTA subdivisions were demarcated by TH immunolabeling.

#### Fluorescence microscopy

We used another group of mice injected with AAV-DJ-ef1α-Cre_ON_/Flp_ON_-ChR2-eYFP, AAV-DJ-ef1α-Cre_ON_/Flp_OFF_-ChR2-eYFP, or AAV-DJ-ef1α-Cre_OFF_/Flp_ON_-ChR2-eYFP for detection of VGluT2 and VGaT in the axon terminals. Coronal LHb sections from mice injected with AAV-DJ-ef1α-Cre_ON_/Flp_ON_-ChR2-eYFP, Coronal nAcc sections from mice injected with AAV-DJ-ef1α-Cre_ON_/Flp_OFF_-ChR2-eYFP, Coronal BNST sections from mice injected with AAV-DJ-ef1α-Cre_OFF_/Flp_ON_-ChR2-eYFP were incubated for 1 h in PB supplemented with 4% BSA and 0.3% Triton X-100. Sections were then incubated with a cocktail of rabbit anti-GFP antibody (GFP-Rb-Af2020; Frontier Institute Co., Ltd, Japan, 1:200), goat anti-VGluT2 antibody (VGluT2-Go-Af1480; Frontier Institute Co., Ltd, Japan, 1:500), guinea pig anti-VGaT antibody (VGAT-GP-Af1000; Frontier Institute Co., Ltd, 1:500) overnight at 4°C. Specificity of primary antibodies has been previous described (Root et al., 2018b). After PB rinsing, sections were incubated in cocktails of fluorescent secondary antibodies (all raised in donkey; Jackson ImmunoResearch Laboratories Inc., 1:100): Alexa Fluor 488 anti-rabbit (711-545-152), Alexa Fluor 594 anti-goat (705-585-147), Alexa Fluor 647 anti-guinea pig (706-605-148). After rinsing, sections were mounted with Vectashield mounting medium (H1000; Vector Laboratories) on slides and air-dried. Fluorescence images were collected with Olympus FV1000 Confocal System (Olympus, Center Valley, PA). High magnification images were taken sequentially with different lasers with 100 × oil immersion objectives and z axis stacks were collected at intervals of 0.2 μm. Imaris microscopy software (Bitplane Inc., South Windsor, CT) was used to analyze z stacks of confocal images from 3 mice for each pathway (62 × 62 × 5 μm for each image) to obtain three-dimensional quantification of axon terminals expressing GFP, VGluT2, or VGaT. The GFP signals were contoured in LHb, accumbens, or BNST. VGluT2 and VGaT immunolabeled fluorescence was masked and the spot tool was used to obtain the number of GFP-expressing terminals that co-expressed VGluT2 alone, VGaT alone, or both VGluT2 and VGaT.

#### Whole-cell electrophysiological recordings

Six to eight weeks after VTA virus injection, mice were anesthetized with isoflurane and perfused with ice-cold, artificial cerebrospinal fluid (ACSF), saturated with 95% O_2_ and 5% CO_2_, and modified to contain (in mM): 92 NMDG, 20 HEPES, 25 glucose, 30 NaHCO_3_,1.2 NaH_2_PO_4_, 2.5 KCl, 5 sodium ascorbate, 3 sodium pyruvate, 2 thiourea, 10 MgSO_4_, 0.5 CaCl_2_. Brains were then removed quickly, placed in this same solution on a VT-1200 vibratome (Leica, Nussloch, Germany), and sectioned through the LHb, nACC, or BNST in coronal slices (200 μm thick). The slices were placed in a holding chamber filled with the same solution, but held at 32°C ACSF. After 15 min, slices were transferred to a holding chamber containing room temperature ACSF modified to contain (in mM): 92 NaCl, 20 HEPES, 25 glucose, 30 NaHCO_3_, 1.2 NaH_2_PO_4_, 2.5 KCl, 5 sodium ascorbate, 3 sodium pyruvate, 2 thiourea, 1 MgSO_4_, 2 CaCl_2_. For recordings, slices were transferred to a chamber superfused 32°C ACSF modified to contain (in mM): 125 NaCl, 2.5 KCl, 1.25 NaH_2_PO_4_, 1 MgCl_2_, 2.4 CaCl_2_, 26 NaHCO_3_ and 11 glucose. Electrodes (4-6MΩ) were backfilled with a Cesium methansulfonate internal solution containing in (mM): 124 CsMeSO_4_, 11 KCl, 0.1 EGTA, 10 HEPES, 10 Na_2_ Phosphocreatine, 4 MgATP, 0.3 Na_2_GTP, 0.5% Biocytin (pH 7.2; 280 mOsm). Cells were visualized on an upright microscope using infrared differential interference contrast video microscopy. Whole-cell voltage-clamp recordings were made using a MultiClamp 700B amplifier (2 kHz low-pass Bessel filter and 10 kHz digitization) with pClamp 10.3 software (Molecular Devices, Sunnyvale, CA). Series resistance (10-30 MΩ) was monitored with a 5 mV hyperpolarizing pulse (50 ms) given every 10 s, and only recordings that remained stable over the period of data collection were used. A 200 μm core optical fiber, coupled to a diode-pumped solid-state laser, was positioned just above the slice and aimed at the recorded cell. Optically-evoked EPSCs or IPSCs were obtained every 10 s with pulses of 473 nm wavelength light (8 mW, 5 ms). EPSCs were recorded at −60 mV while IPSCs were recorded at −0 mV. To determine the monosynaptic nature of neurotransmitter release, TTX (1 μM) followed by 4-AP (200 μM) were bath applied after evoking EPSCs or IPSCs. CNQX (10 μM), Bicuculline (10 μM), or both were added in the perfusion ACSF to block GABA_A_ or Glutamate AMPA receptors respectively. The peak amplitude of EPSCs and IPSCs was measured with the average of 30 consecutive traces.

#### Calcium Imaging

##### Fiber Photometry Recordings and Data Analysis

For all recordings, GCaMP6 was excited at two wavelengths (490nm, calcium-dependent signal and 405 nm isosbestic control) by amplitude modulated signals from two light-emitting diodes reflected off dichroic mirrors and coupled into a 400μm 0.48NA optic fiber. Signals emitted from GCaMP6m and its isosbestic control channel then returned through the same optic fiber and were acquired using a femtowatt photoreceiver (Model 2151; Newport), digitized at 1kHz, and then recorded by a real-time signal processor (RZ5D; Tucker Davis Technologies) running the Synapse software suite. Analysis of the resulting signal was then performed using custom-written MATLAB scripts (linked in methods table). Changes in fluorescence across the experimental session (ΔF/F) were calculated by smoothing signals from the isosbestic control channel (Lerner et al., 2015), scaling the isosbestic control signal by regressing it on the smoothed GCaMP signal, and then generating a predicted 405nm signal using the linear model generated during the regression. Calcium independent signals on the predicted 405nm channel were then subtracted from the raw GCAMP signal to remove movement, photo-bleaching, and fiber bending artifacts. Signals from the GCaMP channel were then divided by the control signal to generate the ΔF/F. Peri-event histograms were then created by averaging changes in fluorescence (ΔF/F) across repeated trials during windows encompassing behavioral events of interest. Video recordings synchronized with neuronal acquisition clocks were acquired at 30 Hz (RV2, TDT).

#### Sucrose Reward Task

Mice were restricted to 85% free-feeding body weight for the duration of the sucrose all behavioral experiments. Two days prior to training, mice were pre-exposed to 8% sucrose in their home cage for 1 hour. One day prior to training, mice were placed in the conditioning chamber and delivered 8% sucrose (40 μl) every 30 s for 30 min. On day 1 of training, mice were placed in the conditioning chamber for 2 h, and a conditioned stimulus, CS+ (7000 Hz, 80 dB, 10 s duration) predicting 8% sucrose was delivered at 0 and 5 s after CS+ onset (20 μl at each delivery). A different conditioned stimulus, CS− (white noise, 80 dB, 10 s duration), did not result in sucrose delivery. Cues were presented with a variable 25-45 s inter-trial interval. Due to scheduling constraints TH-cre mice were trained over two sessions per day. The same training procedure was used for calcium imaging session, except that the CS+ tone resulted in sucrose delivery on 90% of CS+ trials. On 10% of randomly distributed CS+ presentations, the CS+ tone was played without sucrose delivery. These trials were termed sucrose omission trials. The time between the cue onset and the head entry to consume the sucrose was variable for each trial and mouse. To accurately calculate the average peak ΔF/F across trials, we employed a spline-based resampling of the data. This approach is similar to previous methods for aligning variably-timed events (Parker et al., 2016), but resamples the raw data in a time-independent manner, rather than employing predictions derived from a regression model. This approach aligned the tone onset and head entry for sucrose consumption on all trials. A window equal to the duration of the cue→head entry window was sampled before the cue and after the head entry on each trial and all of the data were represented as a proportion of the cue → head entry window latency. These representations of the peak ΔF/F were verified to match the raw data on each trial before sampling specific epochs of time for statistical analysis. The peak ΔF/F for the baseline window was collected from −100% to −50% relative to the cue, the tone window was collected from 0% s to 50% of the cue/head entry window, and the sucrose consumption window was collected from 100% to 150% of the cue/head entry window. These values were then use for statistical testing as described below.

#### Aversive Conditioning

Mice were returned to *ad libitum* feeding following sucrose conditioning and then trained in a classical fear conditioning paradigm. In this task a 5 s, 75 dB, CS+ tone (2,000 Hz) predicted the delivery of a brief, 0.1 s footshock. Mice received a total of 10 cue-shock presentations every 60 s over the course of a 10-minute session. Mice were then tested the subsequent day for freezing responses to the cue in the absence of the shock presentation. Frame by frame timestamps were overlayed onto TDT recorded video files by Avidemux v2.6 and the start and stop of each bout of freezing was recorded for the entire length of the session. Freezing was operationally defined as the absence of all movements with the exception of respiration. The total time spent freezing was then calculated and compared to a group of naive mice that did not receive footshocks during training (tone control group). For the analysis of photometry data, the peak ΔF/F for the baseline window was collected from −5 s to −2.5 relative to the tone, the tone window was collected from 0 s to 2.5 s relative to the tone, and the shock window was collected from 5 s to 7.5 s relative to the tone and used for statistical analysis.

### QUANTIFICATION AND STATISTICAL ANALYSIS

One and two-way ANOVAs were used to compare between group effects, repeated-measures ANOVAs were used to compare within-group effects across time, and a mixed ANOVA was used for analysis with both within- and between-subjects factors. The sphericity assumption for repeated-measures ANOVAs was assessed with the Mauchley sphericity test; when the outcome was significant the *F* values were corrected using the Greenhouse-Geisser approach. The Dunnet’s correction was used for repeated-measures ANOVAs run in prism (whole-cell electrophysiology) and the Sidak correction was used for all other ANOVAs. Statistical analyses were performed with SPSS or Prism. Alpha was always set at p < 0.05.

#### Sample size

The target number of samples in each group for behavioral, anatomical, and electrophysiological experiments was determined on the basis of numbers reported in published studies. The experimenter performing surgeries, including virus vector injection and probe or cannula implantation, was known to hit the targets used with a probability of 0.75. Our target number of animals in each behavioral group was 6-8. We therefore performed about 10–12 surgeries in each group

#### Randomization

All randomization was performed by an experimenter. The same stereotaxic apparatuses were used for all surgeries. All surgical and behavioral manipulations performed on each animal were determined randomly. For animals used in behavioral experiments, the virus used in each animal and injection site were determined randomly and counterbalanced across groups.

#### Exclusion criteria

Data were excluded on the basis of predetermined histological and performance criteria established during pilot experiments. Histological criteria included injection sites and optical fiber or guide cannula placement. Only animals with injection sites in the region of interest were included.

#### Data Availability

Relevant data and code will be made available by the authors upon reasonable request.

## Supplementary Material

1

## Figures and Tables

**Table 1.** VTA Cell-type-Specific Neuronal Activity Profiles during Motivated Behaviors

VTA Cell Type	Reward Cue	Rewarded Port Entry	Reward-Omitted Port Entry	Reward CS−	Reward CS− Port Entry	Unexpected Reward Port Entry	Footshock CS+	Footshock
VGluT2^+^ VGaT^+^	–	↑	–	–	^	↑^a^	–	↑
VGluT2^+^ VGaT^−^	↑	↑	↑	–	↑	–	↑	↑
VGluT2^−^ VGaT^+^	^	↑	↑	↑	↑	–	↑	↑
TH^+^	↑	↑	–	–	–	–^a^	–	↑

–, no change from the baseline activity; ↑, increase in neuronal activity from baseline; ^, moderate increase in neuronal activity. Baseline was defined as the pre-stimulus baseline window with the exception of the unexpected reward port entry, where the baseline is the cued reward port entry.

a VGluT2^+^ VGaT^+^ and TH^+^ neurons show greater responses on cued reward port entry trials and unexpected reward port entry trials, where sucrose was delivered, as compared to reward-omitted port entries, where no reward was delivered.

**Table:** KEY RESOURCES TABLE

REAGENT or RESOURCE	SOURCE	IDENTIFIER
Antibodies		
Mouse anti-mCherry	Takara Bio	Cat# 632543; RRID: AB_2307319
Goat anti-VGluT2	Frontier Institute	Cat# VGluT2-Go-Af310; RRID: AB_2571620
Guinea pig anti-VGAT	Frontier Institute	Cat# VGAT-GP-Af1000; RRID: AB_2571624
Rabbit anti-GFP	Frontier Institute	Cat# GFP-Rb-Af2020; RRID: AB_2571573
Sheep anti-TH	Millipore-Sigma	Cat# AB1542; RRID: AB_90755
Mouse anti-GFP	Takara Bio	Cat# 632381; RRID: AB_2313808
Alexa Fluor 594-AffiniPure donkey anti-mouse	Jackson ImmunoResearch Labs	Cat# 715-585-151; RRID: AB_2340855
Alexa Fluor 647-AffiniPure donkey anti-guinea pig	Jackson ImmunoResearch Labs	Cat# 706-605-148; RRID: AB_2340476
Alexa Fluor 488-AffiniPure donkey anti-rabbit	Jackson ImmunoResearch Labs	Cat# 711-545-152; RRID: AB_2313584
Alexa Fluor 488-AffiniPure donkey anti-mouse	Jackson ImmunoResearch Labs	Cat# 715-545-150; RRID: AB_2340846
Alexa Fluor 594-AffiniPure donkey anti-goat	Jackson ImmunoResearch Labs	Cat# 705-585-147; RRID: AB_2340433
Alexa Fluor 647-AffiniPure donkey anti-sheep	Jackson ImmunoResearch Labs	Cat# 713-605-003; RRID: AB_2340750
Bacterial and Virus Strains		
pAAV-Syn-Flex-GCaMP6s-WPRE-SV40	Addgene	Cat# 100845; RRID: Addgene_100845. Titer: 5 × 10^12^
AAV8-ef1α-C_ON_/F_ON_-mCherry	Karl Deisseroth	RRID: not available
		Titer: 1-3 × 10^12^
AAV8-ef1α-C_ON_/F_OFF_-eYFP	Karl Deisseroth	RRID: not available
		Titer: 1-3 × 10^12^
AAV8-ef1α-C_OFF_/F_ON_-BFP	Karl Deisseroth	RRID: not available
		Titer: 1-3 × 10^12^
AAV8-nEF-C_ON_/F_ON_-hChR2-EYFP	Karl Deisseroth	RRID: not available
		Titer: 5 × 10^12^
AAV8-nEF-C_ON_/F_OFF_-hChR2-EYFP	Karl Deisseroth	RRID: not available
		Titer: 5 × 10^12^
AAV8-nEF-C_OFF_/F_ON_-hChR2-EYFP	Karl Deisseroth	RRID: not available
		Titer: 5 × 10^12^
AAV-DJ-ef1α-Cre_ON_/Flp_ON_-ChR2-eYFP	Stanford University Gene Vector and Virus Core	RRID: not available
		Titer: 5 × 10^12^
AAV-DJ-ef1α-Cre_ON_/Flp_OFF_-ChR2-eYFP	Stanford University Gene Vector and Virus Core	RRID: not available
		Titer: 5 × 10^12^
AAV-DJ-ef1α-Cre_OFF_/Flp_ON_-ChR2-eYFP	Stanford University Gene Vector and Virus Core	RRID: not available
		Titer: 5 × 10^12^
AAV8-ef1α-C_ON_/F_ON_-GCaMP6m	Karl Deisseroth	RRID: not available
		Titer: 3 × 10^12^
AAV8-ef1α-C_ON_/F_OFF_-GCaMP6m	Karl Deisseroth	RRID: not available
		Titer: 3 × 10^12^
AAV8-ef1α-C_OFF_/F_ON_-GCaMP6m	Karl Deisseroth	RRID: not available
		Titer: 3 × 10^12^
Experimental Models: Organisms/Strains		
Mouse: C57BL/6J	The Jackson Laboratory	Cat# JAX:000664; RRID: IMSR_JAX:000664
Mouse: *Slc17a6^tm2(cre)Lowl^*/J (VGluT2-ires-Cre)	The Jackson Laboratory	Cat# JAX:016963; RRID: not available
*Mouse: Slc32a1-IRES2-FlpO-D* (VGaT-ires-FlpO)	The Allen Institute; Daigle et al., 2018	Cat# JAX: 031331; RRID: not available
Mouse: *Th^tm1(cre)Te^*/J (TH-ires-Cre)	International Mouse Strain Resource	MGI:3056580; RRID: not available
Oligonucleotides		
RNAscope probe Mm-Slc17a6	Advanced Cell Diagnostics	Cat# 319171; RRID: not available
RNAscope probe Mm-Slc32a1	Advanced Cell Diagnostics	Cat# 319191-C3; RRID: not available
Software and Algorithms		
GraphPad Prism 8.0	GraphPad Software	RRID: SCR_002798
SPSS	IBM	RRID: SCR_002865
Adobe Photoshop	Adobe Systems	RRID: SCR_014199
Adobe Illustrator	Adobe Systems	RRID: SCR_010279
Statistica 12.0	StatSoft	RRID: SCR_014213
pClamp 10.3	Molecular Devices	RRID: SCR_011323
OriginPro 2017	OriginLab	RRID: SCR_014212
MATLAB	Mathworks	RRID: SCR_001622
Med PC IV	Med-Associates	RRID: not available
MATLAB Scripts	Mathworks	RRID: not available; https://github.com/djamesbarker/FiberPhotometry
Avidemux v2.6	Sourceforge	RRID: not available; http://avidemux.sourceforge.net/

## Highlights

- Accessed glutamate, GABA, and glutamate-GABA neurons in ventral tegmental area (VTA)
- Glutamate neurons and GABA neurons signal reward expectation and learned cues
- Glutamate-GABA neurons signal outcomes, but not their learned predictors
- Glutamate-GABA neurons signal violations in reward expectation
