# Supplementary material for: Distinct Signaling by Ventral Tegmental Area Glutamate, GABA, and Combinatorial Glutamate-GABA Neurons in Motivated Behavior
Source: Cell Rep. Author manuscript; Available in PMC 2020 Oct 14. (PMC7556367; doi:10.1016/j.celrep.2020.108094)
Supplement: 1 [file NIHMS1635132-supplement-1.pdf]

Cell Reports, Volume *32*

Supplemental Information

Distinct Signaling by Ventral Tegmental Area Glutamate, GABA, and Combinatorial Glutamate-GABA Neurons in Motivated Behavior

David H. Root, David J. Barker, David J. Estrin, Jorge A. Miranda-Barrientos, Bing Liu, Shiliang Zhang, Hui-Ling Wang, Francois Vautier, Charu Ramakrishnan, Yoon Seok Kim, Lief Fenno, Karl Deisseroth, and Marisela Morales

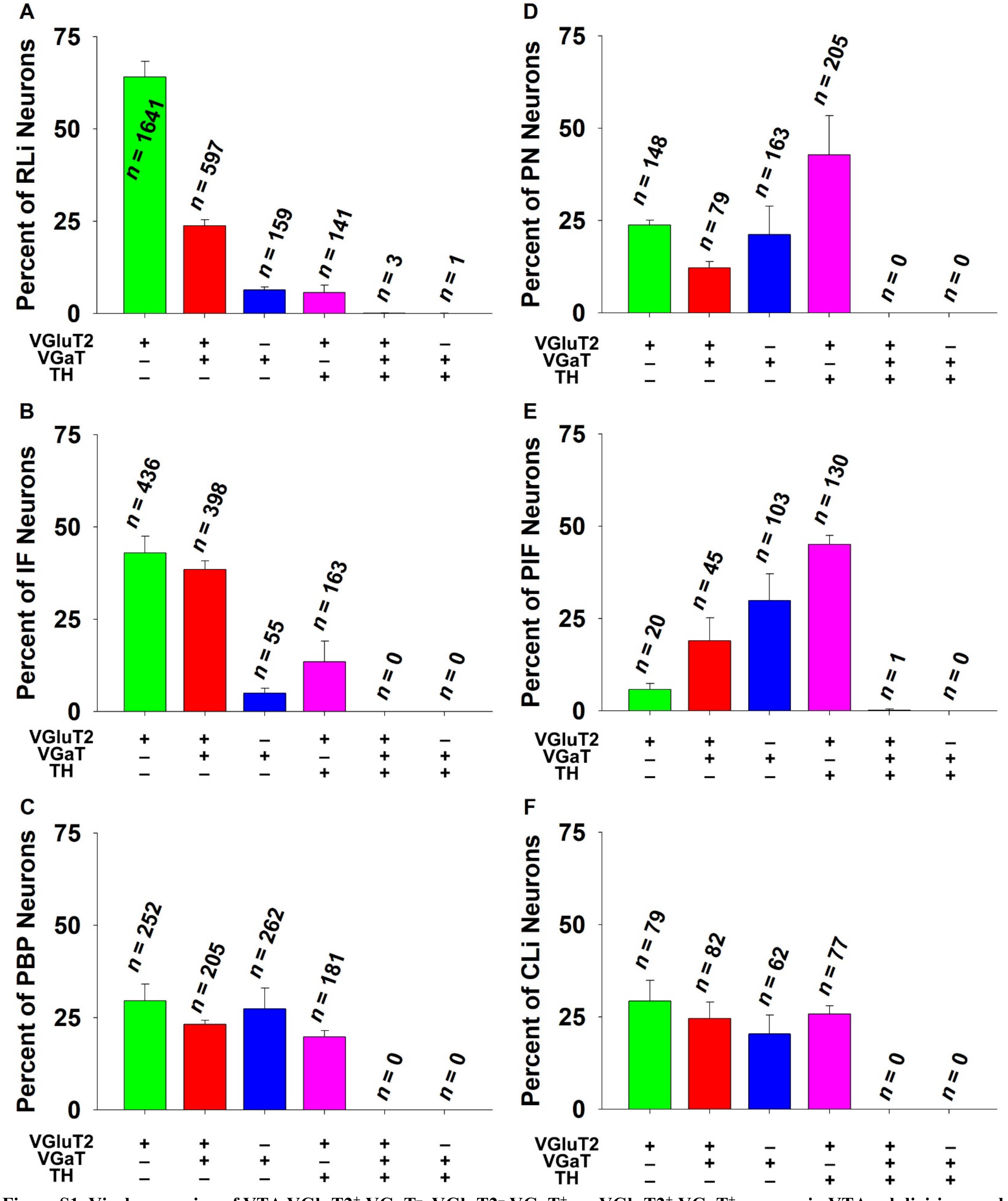

Figure S1. Viral expression of VTA VGluT2⁺ VGaT⁻, VGluT2⁻ VGaT⁺, or VGluT2⁺ VGaT⁺ neurons in VTA subdivisions and their detected co-expression with TH-immunoreactivity. Related to Figure 1. RLi – rostral linear, IF – interfascicular, PBP – parabrachial pigmented, PIF – parainterfascicular, PN – paranigral, PIF – parainterfascicular, CLi – caudal linear. Data are mean ± SEM from 3 mice.

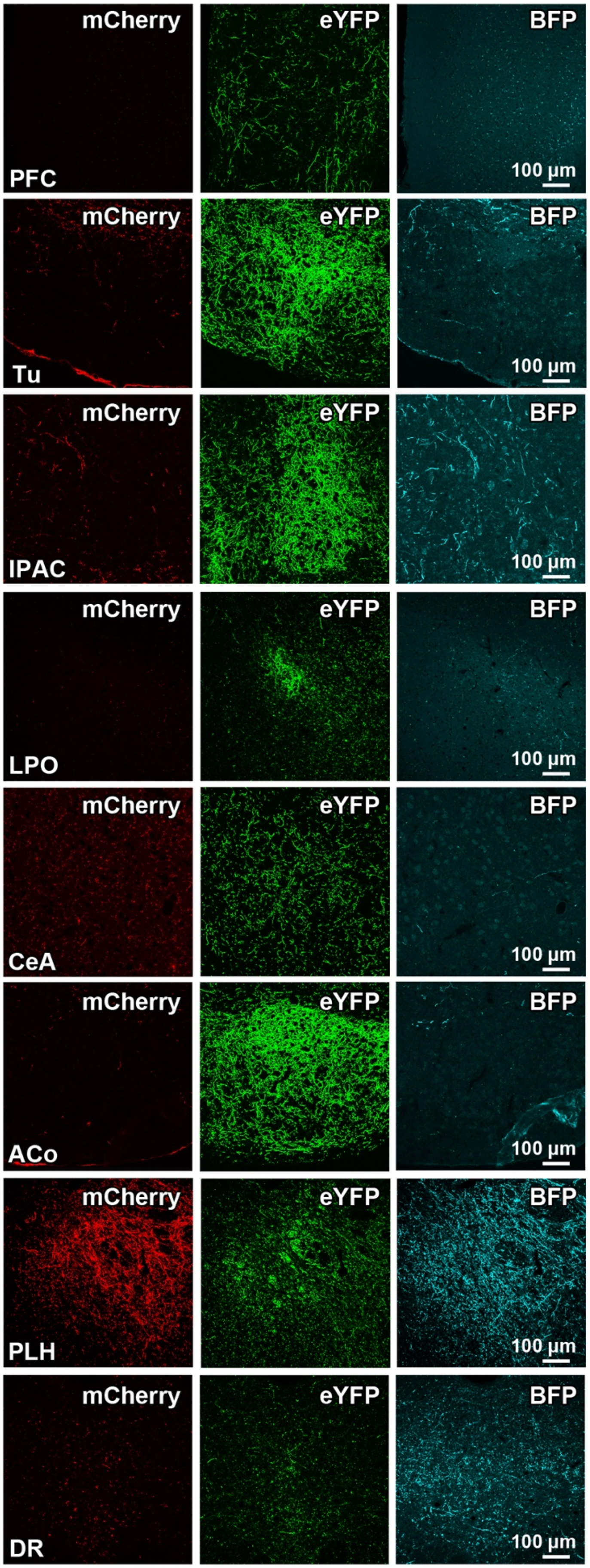
mCherry
eYFP
BFP
PFC
Tu
IPAC
LPO
CeA
ACo
PLH
DR
100 µm

Figure S2. Differential projections of VTA VGluT2^{+} VGaT^{+} neurons (mCherry), VGluT2^{+} VGaT^{-} neurons (eYFP) or VGluT2^{-} VGaT^{+} neurons (BFP). Related to Figure 3. PFC, prefrontal cortex; Tu, olfactory tubercle; IPAC, interstitial nucleus of the posterior limb of the anterior commissure; LPO, lateral preoptic nucleus; CeA, central amygdala; ACo, anterior cortical amygdaloid area; PLH, peduncular part of the lateral hypothalamus; DR, dorsal raphe.

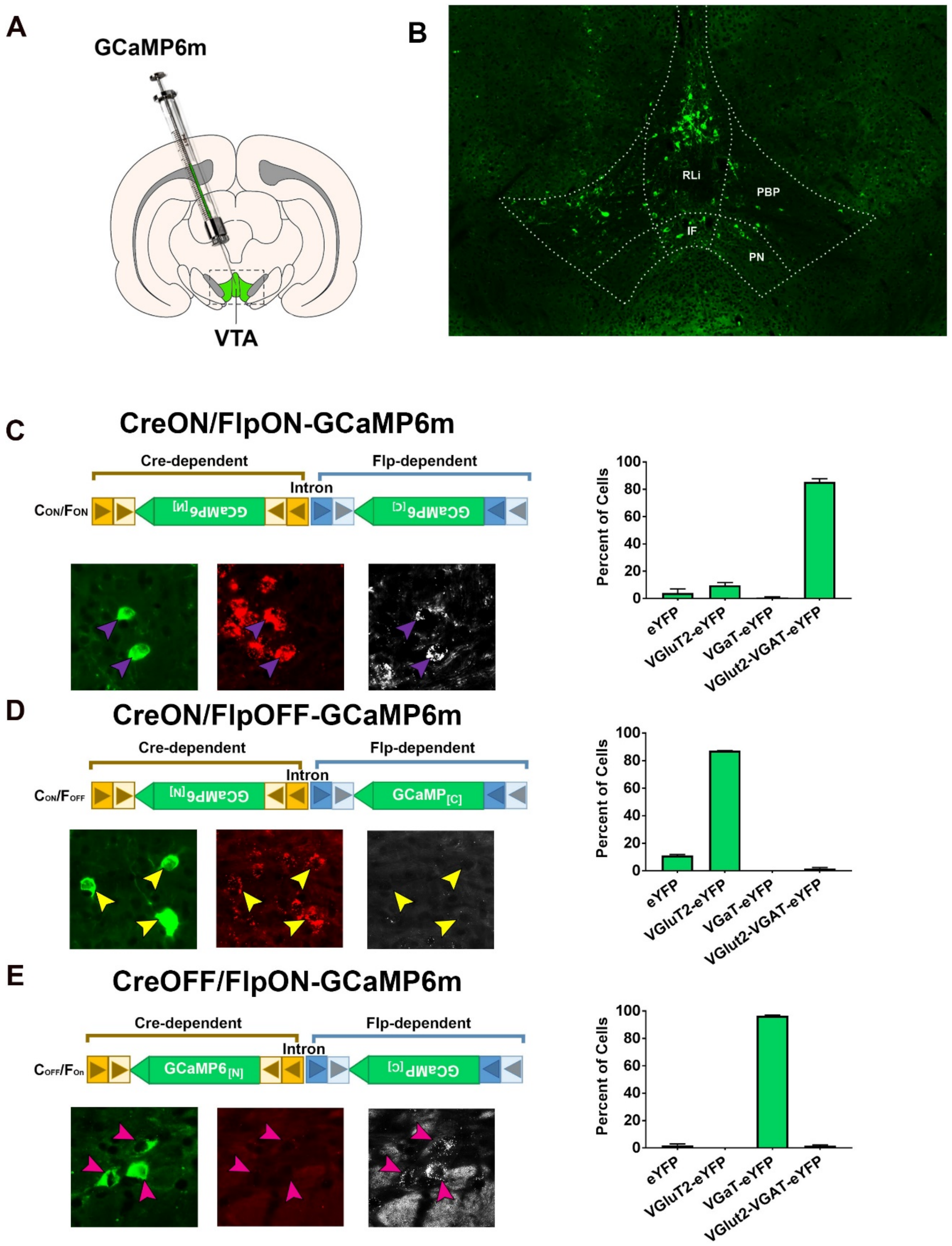

Figure S3. Cell-type specific expression of GCaMP6m. Related to Figures 4-6. (A) Schematic injection of AAV-C$_{ON}$/F$_{ON}$-GCAMP6m (for targeting of VGluT2^{+} VGaT^{+} neurons), AAV-C$_{ON}$/Flp$_{OFF}$-GCAMP6m (for targeting of VGluT2^{+} VGaT^{-} neurons) or AAV-C$_{OFF}$/F$_{ON}$-GCAMP6m (for targeting of VGluT2^{-} VGaT^{+} neurons) into VTA of *vgat-Flp* mice or AAV-DIO-GCaMP6m into the VTA of *th-Cre* mice. (B) Detection of GCaMP6m expression in the VTA with eYFP-immunolabeled GCaMP neurons and *in situ* hybridization. (C) VTA from AAV-C$_{ON}$/F$_{ON}$-GCAMP6m injected mice showing that most GCaMP neurons co-express VGluT2 mRNA and VGaT mRNA. (D) VTA from AAV-C$_{ON}$/F$_{OFF}$-GCAMP6m injected mice showing that most GCaMP neurons express VGluT2 mRNA and lack VGaT mRNA. (E) VTA from AAV-C$_{OFF}$/F$_{ON}$-GCAMP6m injected mice showing that most GCaMP neurons co-express VGaT mRNA and lack VGluT2 mRNA. Data are mean ± SEM.

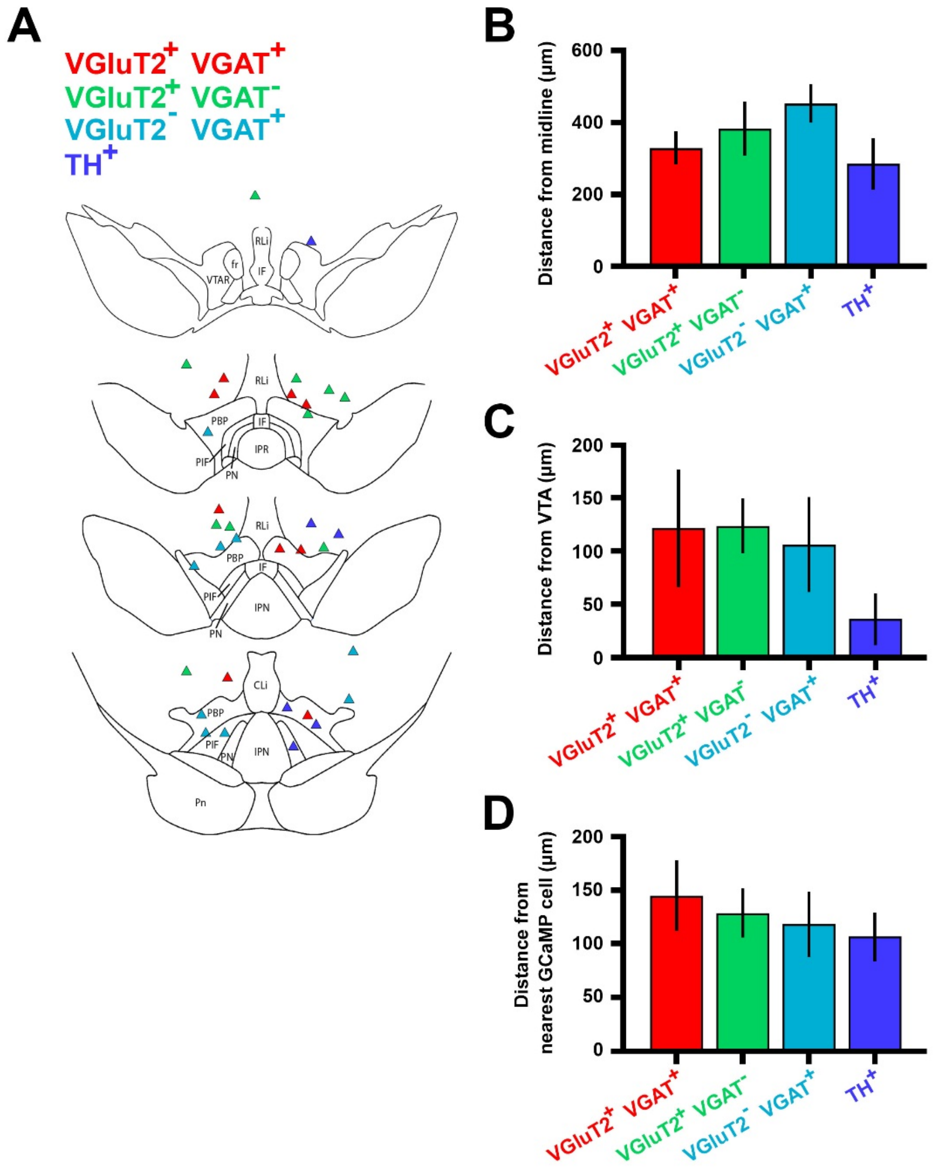

Figure S4. Distribution of optic fibers recording VTA cell-type specific responses. Related to Figures 4-6. (A) Diagram of optical fiber localization in VTA of recorded mice expressing GCAMP in VGluT2^{+} VGaT^{+}, VGluT2^{+} VGaT$^{–}$, VGluT2$^{–}$ VGaT^{+} or TH^{+} neurons. (B) Measurements of the distance between the Midline and the center of implanted fibers over VTA VGluT2^{+} VGaT^{+}, VGluT2^{+} VGaT$^{–}$, VGluT2$^{–}$ VGaT^{+} or TH^{+} neurons. (C) Measurements of the distance from the dorsal border of the VTA and the center of implanted fibers over VTA VGluT2^{+} VGaT^{+}, VGluT2^{+} VGaT$^{–}$, VGluT2$^{–}$ VGaT^{+} or TH^{+} neurons. (D) Measurements of the distance between the nearest identified GCaMP6m positive cell and optic fibers implanted over VTA VGluT2^{+} VGaT^{+}, VGluT2^{+} VGaT$^{–}$, and TH+ neurons.

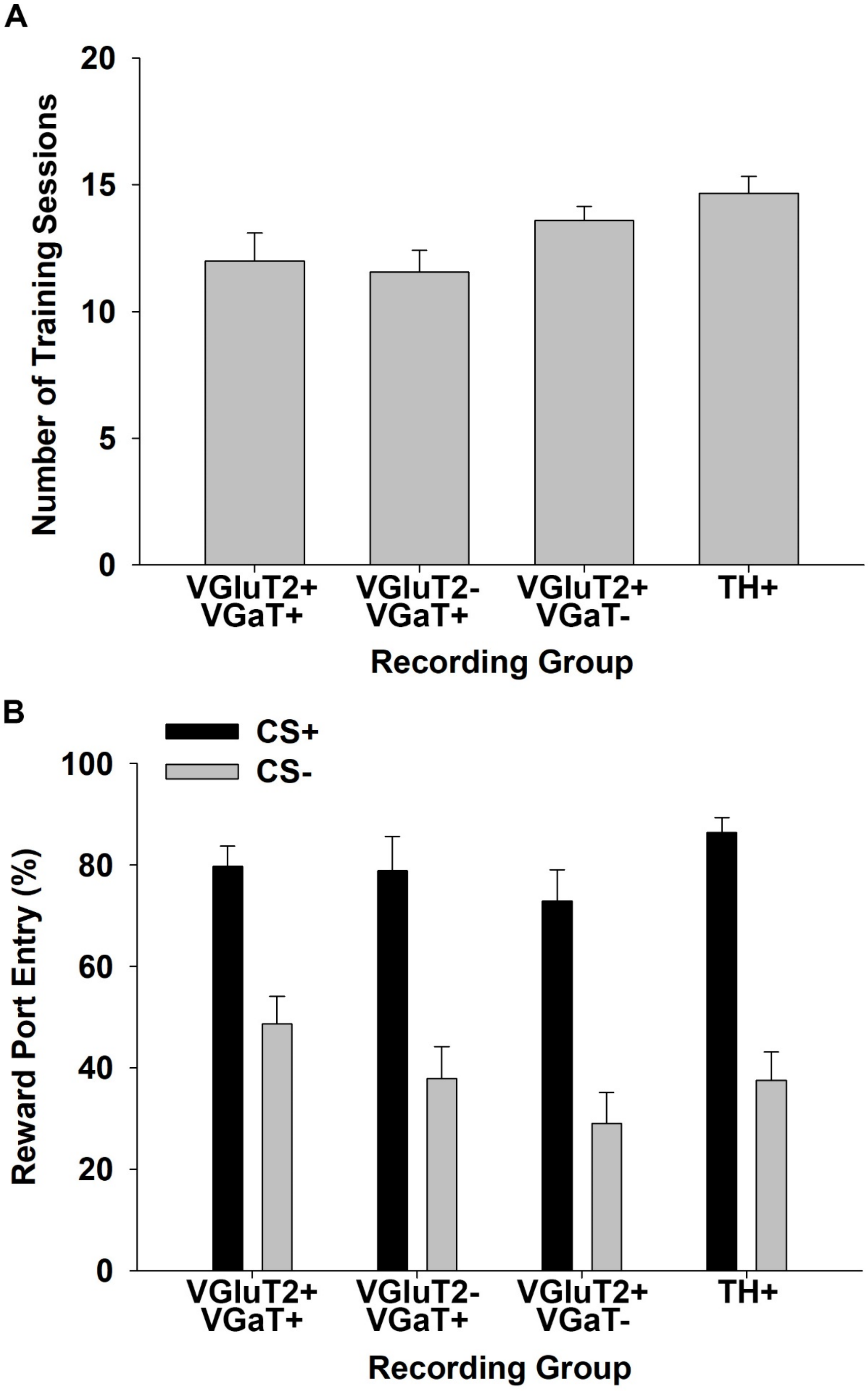

Figure S5. Mice discriminate CS+ reward cues from CS- non-reward cues. Related to Figure 4. (A) Number of training days did not differ by group, $F_{(3,29)} = 2.797$, 0 = 0.058. (B) Percent of reward port entries during CS+ or CS- trials during the reward-seeking task. No effect of group, $F_{(3,29)} = 1.65$, $p = 0.199$, no cue x group interaction, $F_{(3,29)} = 0.95$, $p = 0.428$, but main effect of cue, $F_{(1,29)} = 127.62$, $p < 0.001$. Sidak-adjusted pairwise comparison indicated significantly higher percent of reward port entries following CS+ compared to CS- across all groups, $p < 0.001$. Data are mean ± SEM. ** $p < 0.01$, *** $p < 0.001$.

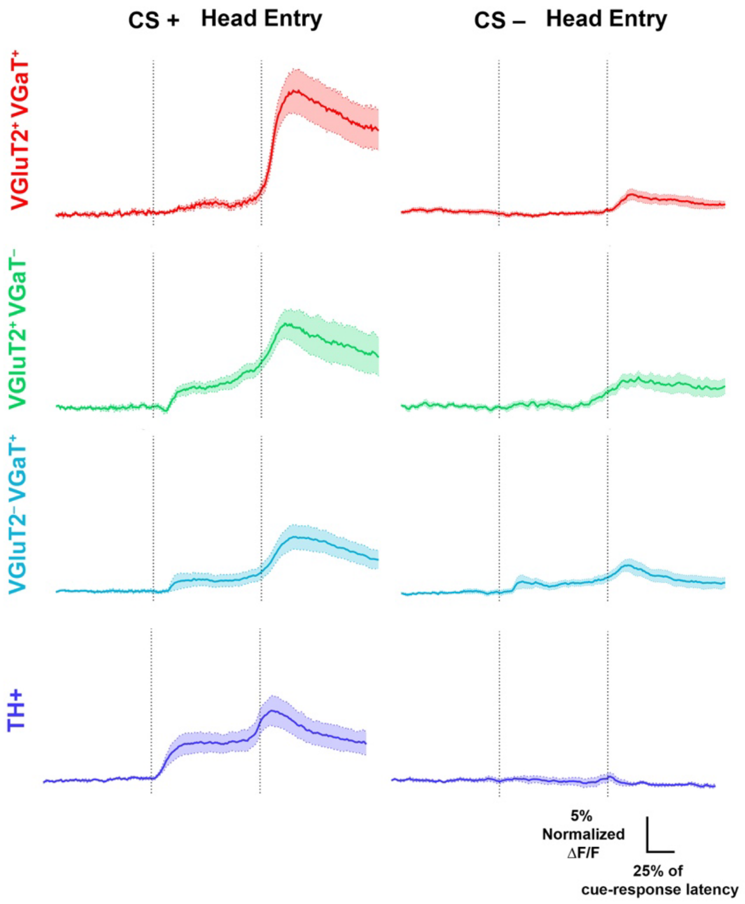

Figure S6. Temporally aligned responses to the CS+ or CS- and head entry during sucrose-seeking behavior. Related to figure 4. Average of cell population Ca^{2+} signals by VTA cell-types (VGluT2^{+} VGaT^{+}, VGluT2^{+} VGaT$^{–}$, VGluT2$^{–}$ VGaT^{+}, and TH^{+}) during sucrose seeking in response to CS+ (Left) or CS – (Right). VGluT2^{+} VGaT^{+} neurons did not show Ca^{2+} signal increases in response to cues. VGluT2^{+} VGaT$^{–}$ and TH^{+} neurons increased their Ca^{2+} signals in response to cues predicting reward (CS+) (VGluT2^{+} VGaT$^{–}$, $p<0.01$; TH+, $p<0.001$). VGluT2$^{–}$ VGaT^{+} neurons showed a moderate increase in Ca^{2+} signal in response to both CS+ ($p=0.064$), but a significant increase to the cue predicting the absence of reward (CS–) ($p=0.041$). All VTA cell types increased their Ca^{2+} signal at the time of head entry to consume the reward (VGluT2^{+} VGaT$^{–}$, $p<0.001$; VGluT2$^{–}$ VGaT^{+} $p<0.01$; VGluT2^{+} VGaT^{+}, $p<0.001$; TH+, $p<0.01$). Among all types of VTA neurons, the TH^{+} neurons did not show increases in Ca^{2+} signal during head entries following the CS- (Baseline vs. Head-Entry during CS-: VGluT2^{+}VGaT^{+}, $P<0.05$, VGluT2^{+} VGaT$^{–}$, $p<0.001$; VGluT2$^{–}$VGaT^{+}, $p<0.05$;). Excepting VGluT2$^{–}$ VGaT^{+} neurons, the different VTA cell types differentiated between the CS+ and CS- (VGluT2^{+} VGaT$^{–}$, $P<0.01$; VGluT2^{+}VGaT^{+}, $p<0.001$; TH^{+}, $p<0.01$) [N=6-10 per group; Mixed ANOVA; Group × Cue-Type × Event interaction; $F_{(12,108)}=4.802$, $P<0.0001$; Sidak-correction for pos-hoc comparisons].

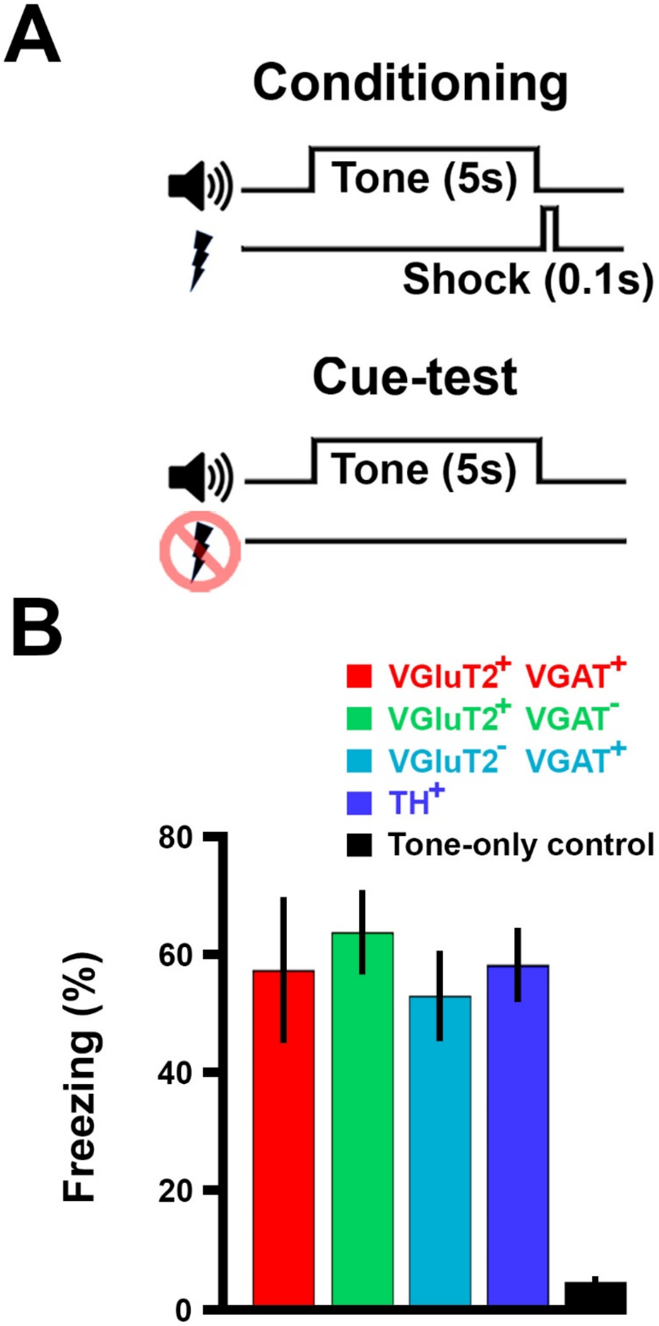

Figure S7. Mice freeze in response to cues associated with footshock. Related to Figure 6. (A) Schematic of fear-conditioning training and testing procedures. (B) Increases in freezing behavior in conditioning footshock mice in response to the cue (tone) as compared with mice that never received footshocks [$F_{(4,28)}$ =10.275, p<0.001; Sidak-adjusted post hoc all p<0.01].
